# An integrated wireless deep-UV sensing system for intelligent early fire detection

**DOI:** 10.1126/sciadv.aef4143

**Published:** 2026-05-27

**Authors:** Taehyun Park, Junhwa Oh, Junhyung Cho, Seungme Kang, Nicola Gasparini, Wooseok Song, Jaehyun Hur, Garam Bae, Seyong Oh, Hocheon Yoo

**Affiliations:** ^1^ Department of Electronic Engineering, Hanyang University, Seoul 04763, Republic of Korea.; ^2^ Division of Electrical Engineering, Hanyang University ERICA, 55 Hanyangdaehak-ro, Ansan 15588, Republic of Korea.; ^3^ Department of Artificial Intelligence Semiconductor Engineering, Hanyang University, 222 Wangsimni-ro, Seoul 04763, Republic of Korea.; ^4^ Department of Chemistry and Centre for Processable Electronics, Imperial College London, London SW7 2AZ, UK.; ^5^ Thin Film Materials Research Center, Korea Research Institute of Chemical Technology, Daejeon 34114, Republic of Korea.; ^6^ School of Electronic and Electrical Engineering, Sungkyunkwan University, Suwon 16419, Republic of Korea.; ^7^ School of Chemical, Biological, and Battery Engineering, Gachon University, Gyenggi 13120, Republic of Korea.; ^8^ Department of Physics, Dankook University, 119 Dandae-ro, Cheonan 31116, Republic of Korea.

## Abstract

Uncontrolled fires, from wildlands to industrial facilities, have become a pressing global threat, causing widespread ecological damage, loss of life, and economic disruption. The fundamental challenge is the lack of rapid and reliable detection at the ignition stage. Once flames spread, suppression becomes increasingly difficult and damage escalates. Conventional methods such as smoke detectors and thermal imaging fall short for wildfire and large-scale fire scenarios. Effective systems require accurate detection without false activation, stable performance under varied and harsh environments, and low power or zero-bias photodetection for continuous use in remote locations. Here, we report a wireless and flexible deep-ultraviolet (DUV) sensing platform that addresses these requirements in a single integrated unit. The platform combines a zinc tin oxide nanocomposite photodetector, flexible circuit integration, portable power, and Bluetooth communication. The sensor shows a selective solar-blind DUV response, stable operation under mechanical stress and extended cycling (92.5% retention after 100 bending cycles and 96.7% after 180 days), and energy-efficient performance compatible with autonomous deployment. Data-driven analysis of response curves allows for machine learning models that classify flame types and estimate distance, extending the system beyond binary fire detection by providing additional information on flame type and relative distance. This integrated approach provides a practical route to reliable fire monitoring, relevant to early-stage fire monitoring concepts for wildfire and industrial safety applications.

## Teaser

A solar-blind deep-UV platform combines nanocomposite photodetectors with flexible electronics and analytics for flame monitoring.

## INTRODUCTION

Wildfires and large-scale fires are occurring with increasing frequency and severity, driven by climate change and extended periods of heat and drought (*1*, *2*). These events cause ecological destruction, human casualties, and economic loss, and they are now regarded as persistent systemic threats rather than short-term irregularities. The central challenge is delayed detection. Once ignition progresses beyond the initial stage, flames spread rapidly, and if the timing of discovery is missed, the fire often grows uncontrollably until it extinguishes naturally (*3*, *4*). Early and reliable detection before fire propagation is therefore essential (*5*–*7*). Detection must not only confirm the presence of a flame but also identify its source and combustion characteristics, given that these factors determine how the fire will evolve and how it should be controlled. For example, suppression tactics and the recommended extinguishing agent can be strongly fuel-dependent, so early identification of the combustion source can support prompt selection of an appropriate response strategy (*8*, *9*). In addition, source-type information enables more informed decision-making and prioritization, which can reduce secondary damage and improve the efficiency of suppression efforts in the early stage (*10*).

Conventional fire detection technologies, such as smoke detectors (*11*, *12*) and thermal imaging cameras (*13*, *14*), have intrinsic limitations. Smoke detectors rely on the inflow of particles, which delays their response and reduces reliability in open areas (*15*). Thermal infrared cameras can provide real-time imaging, but their coverage is narrow, false alarms are frequent, and cost limits their deployment at scale (*16*). These shortcomings emphasize the need for a detection method that is rapid, selective, and deployable across diverse outdoor conditions. For application in wildfires and large-scale fires, several technical requirements are clear. Accurate detection is essential, meaning that false responses caused by noise must be eliminated (*15*, *17*). Long-term stability is required to maintain performances in varied and harsh environments (*18*). Energy demand must be minimized, with low-power operation (*19*) or self-sustained functionality (*20*) preferred for extended use in wildfire monitoring and large industrial facilities.

Deep-ultraviolet (DUV) photodetectors (PDs) offer a direct solution. Radiation in the range of 200 to 280 nm is absorbed almost completely by the ozone layer (*21*, *22*), so signals measured at the ground level originate primarily from combustion (*23*, *24*) and other high-energy reaction phenomena (*25*). This spectral property enables detection of flames with high specificity and essentially no environmental interference. The response occurs at ignition, allowing recognition at the earliest stage (*16*). Most reported systems remain rigid, depend on external power, or require separate processing units (*26*–*28*). These limitations restrict portability and autonomy, which prevents broad use in wildfire and large-scale fire monitoring.

Here, we present a fully integrated, low-power DUV sensing platform that unites detection, circuit operation, and wireless communication in a single flexible unit. The core element is a zinc tin oxide (ZTO) (*29*–*32*) nanocomposite layer prepared by dispersing hydrothermally synthesized nanoparticles (NPs) into a sol-gel matrix. This composition produces uniform and processable films while retaining the 3.97 eV of wide bandgap required for a selective DUV response. The photodetector is directly coated on a flexible printed circuit, enabling circuit-level integration and conformal attachment to various surfaces. The platform operates with a portable power supply and a Bluetooth link that transmits sensing data to mobile devices in real time, allowing autonomous use without external infrastructure. The design addresses key technical requirements for wildfire and large-scale fire detection. A selective DUV response ensures accurate sensing with no false activation from environmental noise. The nanocomposite film and flexible circuit structure retain 92.5% of its initial performance after 100 repeated bending cycles and 96.7% of the photocurrent after 180 days of storage, indicating robust durability and long-term stability under outdoor-relevant conditions. Low power consumption and the capacity for zero-bias photodetection support continuous use in remote locations where frequent maintenance is impractical. Alongside the hardware, data-driven analysis of DUV response curves from different flame sources trains machine learning (ML) models to classify flame type relative intensity and estimate distance, extending functionality beyond binary detection.

## RESULTS

**Fig. 1.**
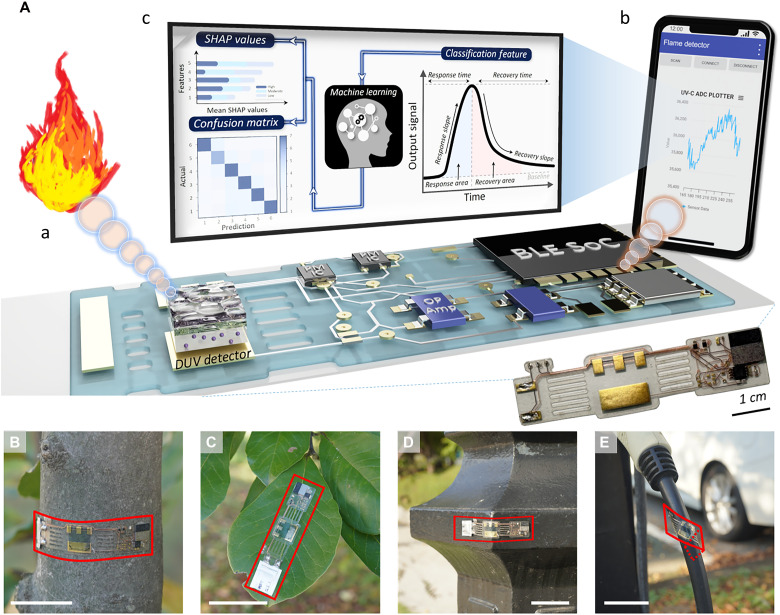
Conceptual diagram of the circuit-integrated DUV photodetectors (PDs) for flame sensing and ML-connected classification framework. (**A**) Schematic illustration representing the concept of this work: (a) DUV-induced flame signal sensing with low environmental background noise, (b) circuit-integrated sensor system enabling wireless signal transmission and low power operation, and (c) ML-based estimation of flame source, distance, and intensity. A photograph of the fabricated flexible sensor-board device is shown as an inset, with a 1-cm scale bar for size reference. Photographs of the proposed circuit-integrated ZTO nanocomposite DUV PDs attached on various surfaces including (**B**) tree bark, (**C**) leaves, (**D**) steel structures, and (**E**) a power cable. Scale bars represent 5 cm.

Figure 1A summarizes the overall framework and the design rules that guided the development of this system. Building on the challenges outlined earlier, we designed the sensing platform with four guiding principles. The first target was to achieve a selective DUV response, ensuring that the detector responds primarily to flame-origin ultraviolet radiation while rejecting visible light. This was realized using a ZTO nanocomposite with a wide bandgap, formulated through a solution process and deposited directly onto a printed circuit substrate. The second target was to secure mechanical stability in flexible form so that the sensor can operate reliably when mounted on nonplanar or irregular surfaces such as bark, leaves, or steel structures (Fig. 1, B to E). The third target was system-level integration with wireless communication, embedding the signal conditioning, microcontroller, power source, and Bluetooth module in the same flexible unit to eliminate the need for bulky external equipment. The fourth target was to incorporate ML for flame classification, enabling the construction of a training database from DUV response curves and extending the device beyond binary fire detection toward predictive situational awareness.

**Fig. 2.**
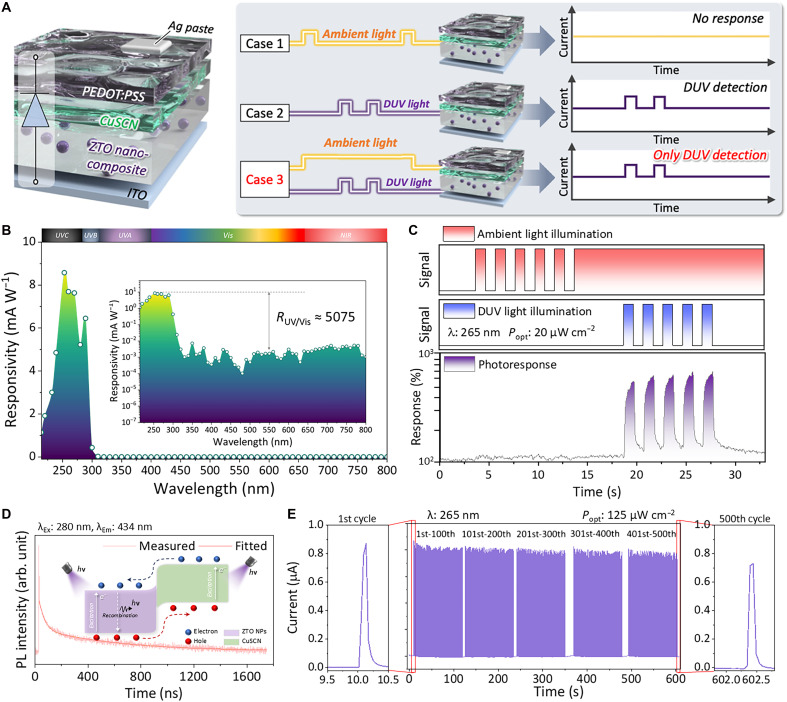
DUV sensing performance of the unit ZTO nanocomposite–based DUV PD. (**A**) Schematic illustration of the DUV PD representing environmental (ambient) light interference resilience DUV sensing. (**B**) Spectral responsivity of the proposed DUV PD. The inset displays log-scaled spectral responsivity with UV/visible rejection ratio. (**C**) Transient response of the DUV PD under DUV illumination with dark and ambient light condition. (**D**) Time-resolved photoluminescence (PL) characterization results of the ZTO/CuSCN heterojunction film indicating long carrier lifetime. (**E**) Transient photoresponse of the DUV PD during repeated 500 cycles.

To achieve a flexible DUV photodetector with high charge transport, we developed a ZTO nanocomposite approach. Hydrothermally synthesized ZTO NPs were blended into a ZTO sol-gel matrix, and the mixture was spin-coated to form uniform thin films. This nanocomposite strategy uses the high crystallinity and wide bandgap of the ZTO NPs, while the sol-gel network provides good interparticle connectivity (*33*), mitigating the poor charge mobility typically found in NP-only films owing to the electron hopping mechanism (*34*). The NP-embedded film is continuous and defect-minimal, as evidenced by electron microscopy and elemental mapping (figs. S1 and S2), with a thickness of 65 nm (cross section in fig. S3). The crystalline properties of the NPs were confirmed by x-ray diffraction, where peaks at 26.51°, 34.3°, 36.2°, and 51.8° correspond to the orthorhombic ZTO (012), (104), (015), and (214) planes (fig. S4) (*31*, *35*). The film’s optical absorption spectrum and corresponding energy bandgap extracted from the Tauc plot (fig. S5) display an absorption edge corresponding to a wide optical bandgap of 3.97 eV. This wide bandgap aligns with the solar-blind DUV range (200 to 280 nm), which suppresses visible-light sensitivity and enables preferential response to DUV photons. To evaluate the validity of the ZTO nanocomposite–based DUV photodetector before circuit integration, we fabricated the unit photodetector with a layered architecture on a flexible substrate, as displayed in Fig. 2A. An indium tin oxide (ITO) layer on polyethylene terephthalate (PET) serves as the bottom electrode. The ZTO nanocomposite film was deposited on the ITO, and atop this, we added a layer of copper(I) thiocyanate (CuSCN). Last, a poly(3,4-ethylene dioxythiophene)-poly(styrenesulfonate) (PEDOT:PSS) layer was applied as the transparent top electrode, completing the device stack. The n-type ZTO and p-type CuSCN form a heterojunction with a type II band alignment, which is designed to facilitate charge separation and to increase charge carrier lifetime under DUV illumination. Both ZTO (3.97 eV) and CuSCN (3.5 eV) (*36*) exhibit wide bandgaps, rendering the device effectively insensitive to visible and solar UVA light. The use of PEDOT:PSS on top provides a flexible, transparent electrical contact that conforms to the underlying layers. This fully solution-processed device structure can be implemented directly on flexible circuit boards without high-temperature steps, an important advantage for integration with plastic substrates.

The ZTO nanocomposite photodetector exhibits a solar-blind spectral response, meaning that it is highly responsive to DUV wavelengths while showing negligible responsivity to longer wavelengths such as ambient light (Fig. 2, A and B). Figure 2B shows the spectral photoresponsivity of the proposed device. A pronounced peak responsivity of 8.58 mA W^−1^ is observed at 250 nm (within the DUV band). In contrast, for wavelengths beyond 300 nm, the responsivity drops by several orders of magnitude to negligible levels. This sharp cutoff confirms that the device is selective to the solar-blind UV range. At the ground level, solar irradiance in the 200- to 280-nm band is negligibly small because of strong atmospheric absorption, so the ambient background in the solar-blind DUV window is expected to be near zero (*37*, *38*). A simple modeling case further indicates that ∼97% of ground-level solar irradiance is distributed over 300 to 3000 nm, implying that irradiance in the 200- to 280-nm band is orders of magnitude smaller than the ambient bands (*39*). As a result, under normal illumination conditions (room lights or sunlight-mimicking conditions), the photodetector delivers virtually no photocurrent above the dark level, whereas it responds strongly when a DUV signal is present. The relevant flame-induced DUV signals can be detected with a high signal-to-noise ratio, essentially free from background interference. We verified the above-described hypothesis by testing the device under ambient light. Here, a low DUV irradiance (20 μW cm^−2^) was used to validate switching under conservative conditions and to assess background/ambient-light influence. As represented in Fig. 2C and movie S1, no false response was detected until a DUV light-emitting diode (LED) (265 nm) was turned on, at which point the photocurrent immediately increased. The on/off ratio between 265-nm illumination and no illumination (even in ambient light) exceeded 584%, demonstrating the inherent advantage of solar-blind detection for flame sensing. To further validate operation under a standardized strong-illumination condition, we additionally tested the photodetector under AM1.5G solar-simulator illumination (1 sun). As shown in fig. S6, no discernible switching was observed under AM1.5G illumination, and the AM1.5G-induced photocurrent was only 3.16% of the photocurrent generated under 265-nm DUV excitation, supporting solar-blind operation under sunlight-mimicking conditions. These results suggest that our ZTO/CuSCN photodetector can operate in real-world lighting conditions without sacrificing sensitivity or raising false alarms resulting from the environmental ambient lights.

Next, we further investigated the optoelectronic characteristics of the proposed device. For *I*-*V* (current-voltage relation) characterization, a higher DUV irradiance (496 μW cm^−2^) was used to obtain a clear voltage-dependent photoresponse with a sufficient signal margin (fig. S7A). As displayed in fig. S7A, DUV light–induced 7 mV of open circuit voltage (*V*_oc_) shifts was observed at the given conduction, representing that the working principle of the proposed device is based on the photovoltaic effect, enabling self-powered operation (*40*, *41*). Response speed was evaluated under the nominal irradiance condition (125 μW cm^−2^) to enable a direct comparison across experiments (fig. S7B). The device exhibited a rise time of 71 ms and a decay time of 154 ms (defined from 10 to 90% of peak photocurrent for rise and vice versa for decay). In addition, to further quantify the dynamic response of the device beyond the single-shot rise/decay times, we measured the frequency-dependent photoresponse under input DUV modulation (*42*). The normalized response amplitude gradually decreased with increasing modulation frequency, and the device showed a −3-dB cutoff frequency of ∼185 Hz (fig. S8). This result indicates that the proposed device provides millisecond-scale response dynamics, which is sufficient for the intended flame-sensing scenario where the UV emission persists over seconds and exhibits low-frequency fluctuations rather than submillisecond flashes. To examine how the output scales with incident DUV irradiance and to assess approximate linearity over the operating range, we performed a controlled light-intensity test. This measurement also provides a consistent reference for cross-comparison with subsequent flame-source responses. The device exhibited a clear photocurrent response that increased stepwise with the incident DUV optical power density (fig. S9A). A log-log plot of the photocurrent versus optical power revealed a nearly linear relationship with a power-law exponent of 1.1, confirming quantitative and proportional photoresponse characteristics (fig. S9B). To further investigate the photoinduced charge dynamics at the ZTO and CuSCN heterojunction, time-resolved photoluminescence (PL) measurements were conducted on both pristine ZTO and ZTO/CuSCN bilayer structures (Fig. 2D and figs. S10 and S11). The photoluminescence decay profiles were fitted using a biexponential decay model described by the equation (*43*)

$$I\left(t\right)=A_{1}e^{-t/\tau_{1}}+A_{2}e^{-t/\tau_{2}}$$ (1)

where *A*_1_ and *A*_2_ are the pre-exponential coefficients representing the relative contributions of fast and slow decay pathways, respectively. τ_1_ and τ_2_ correspond to the associated carrier lifetimes. The fast component (τ_1_) is commonly attributed to surface or interface recombination, while the slow component (τ_2_) reflects recombination within the bulk or in trap-limited states (*44*). To obtain a comprehensive measure of the overall carrier dynamics, the average carrier lifetime (τ_ave_) was calculated using the amplitude-weighted formula (*45*)

$$\tau_{\text{ave}}=\frac{A_{1}\tau_{1}^{2}+A_{2}\tau_{2}^{2}}{A_{1}\tau_{1}+A_{2}\tau_{2}}$$ (2)

As shown in table S1, the pristine ZTO film exhibited a fast decay component of τ_1_ = 24.35 ns with *A*_1_ = 41.34 and a slower component of τ_2_ = 386.53 ns with *A*_2_ = 4.19, resulting in an average carrier lifetime of 247.6 ns. In contrast, the ZTO/CuSCN heterojunction showed τ_1_ = 33.53 ns with *A*_1_ = 25.9 and τ_2_ = 481.89 ns with *A*_2_ = 3.63, yielding a longer average carrier lifetime of 333 ns. These results indicate a clear enhancement in carrier lifetime within the heterojunction structure. The longer τ_1_ and τ_2_ observed for ZTO/CuSCN, along with the increased average lifetime, suggest that the introduction of the CuSCN layer suppresses fast surface recombination and promotes bulk carrier retention (*46*, *47*). This behavior is attributed to the built-in electric field formed at the ZTO/CuSCN interface, which enhances the spatial separation of photogenerated electron-hole pairs and reduces nonradiative recombination losses (*48*). Such improved charge dynamics support the photovoltaic behavior observed under DUV illumination and enable the self-powered operation of the device.

For outdoor target use, reliable operation is essential. We investigated the device stability by monitoring its performance over time and under continuous operating conditions. For repeatable extraction of device metrics, we selected a nominal operating irradiance of 125 μW cm^−2^ (265 nm) and evaluated cycle stability under this representative condition. Figure 2E displays the photocurrent response under 265-nm light repeatedly switched on and off for 500 cycles. The ZTO DUV photodetector maintained a consistent on-current and a stable off-current (dark current) throughout these cycles. At the 500th cycle, the photocurrent retained 84% of its initial value. Further evaluation was conducted in terms of storage stability. As represented in fig. S12, after 180 days at the first measurement, the photocurrent remained at an average of 96.7% among five cycles. For other key parameters, the dark current changed slightly from 7.52 to 8.66 nA, and the rise/decay times showed only a minor change, with a difference of a few milliseconds from the pristine state. Specifically, in its pristine state, the device exhibited rise and decay times of 62.32 and 107.23 ms, respectively. After 180 days, they decreased to 56.82 and 91.65 ms, respectively.

**Fig. 3.**
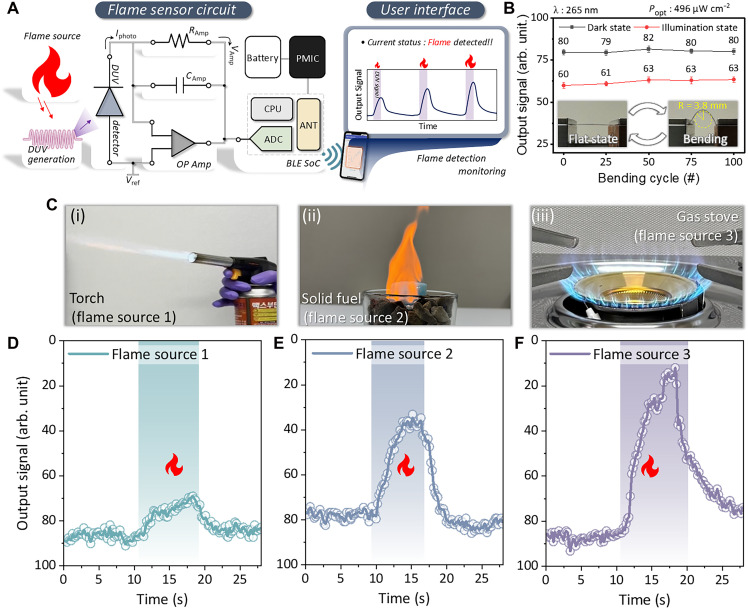
Circuit-integrated ZTO DUV PD: Mechanically robust DUV sensing performance and flame sensing performance. (**A**) Schematic illustration of the FPCB with a detail description of the circuit units, including operational amplifier (OP Amp), BLE SoC, and detector. The circuit-integrated ZTO nanocomposite DUV PD was directly fabricated on the FPCB. In this case, the bottom Au electrode was preformed at the FPCB surface. The ZTO nanocomposite, CuSCN p-type layer, and top PEDOT:PSS electrode were fabricated through spin coating. The top PEDOT:PSS electrode was connected to a predefined circuit electrode line. (**B**) Operation stability represented by the scatter line plot according to the flat-bending cycle. (**C**) Photographs of the flame sources used in the real-time flame sensing measurement: (i) torch, (ii) solid fuel, and (iii) gas stove. Measured sensor system signals under (**D**) torch, (**E**) solid-fuel, and (**F**) gas-stove flame irradiation. The specific distance between the sensor system and flame sources was 40 cm. The shaded (gradient) region uniformly denotes the flame-on interval, during which the flame source was present.

Figure 3A illustrates the design of the flexible printed circuit board (FPCB) that hosts not only the ZTO photodetector but also the necessary electronic components for signal processing and wireless communication. The photodetector is mounted onto the FPCB and electrically connected to a transimpedance amplifier on the basis of an operational amplifier. This analog front end converts the photodetector’s photocurrent into a voltage signal with appropriate gain and filtering. The conditioned signal is then fed into a Bluetooth Low Energy (BLE) system on chip (SoC) on the same board. The BLE SoC digitizes the signal and handles wireless data transmission. The entire assembly is powered by a compact battery, making the sensor unit completely portable. Because all components are integrated on a thin PET substrate, the system is mechanically flexible and ultralightweight. We demonstrated the conformability by attaching the sensor unit to various curved and irregular surfaces relevant for fire monitoring, such as a tree trunk, plant leaves, and power cables (Fig. 1, B to E). To evaluate the stable device operation on the above-described various bending conditions, we quantitatively investigated the bending resilience of the integrated sensor system. As displayed in fig. S13, the circuit-integrated ZTO photodetector exhibited a similar response curve shape to that of a unit photodetector (fig. S9A). The device was bent to different radii (ranging from flat to 3.1 mm) and held in that state while measuring its photoresponse to a constant DUV illumination (fig. S14). Because mechanical deformation can introduce additional variability in the readout, bending-cycle tests were conducted at a higher DUV irradiance (496 μW cm^−2^ at 265 nm) to ensure a robust signal-to-noise ratio and reliable tracking. We found no notable loss of photocurrent or change in response speed even at the smallest bending radius, indicating that the photodetector and circuit remain operational under substantial flexure. Next, repeated bending tests were conducted where the sensor was cyclically flexed (e.g., 100 cycles under a bending radius condition of 3.8 mm). As shown in Fig. 3B, the photocurrent of the device after various bending cycles stays within 92.5% of its initial value under the same illumination condition. The scatterplot of response versus bending cycles/radius exhibits no obvious degradation trend. These results demonstrate that the sensor can withstand the mechanical demands of real-world use, such as being mounted on moving objects or transportation, with minimal performance loss.

In addition to mechanical robustness, regarding outdoor operation, we quantitatively evaluated the environmental stability of the flexible sensor-board device under controlled variations in temperature, humidity, ambient composition, and pressure. First, temperature-dependent measurements were performed from 20° to 100°C (in 20°C increments), and the transient response was recorded under 0.5-Hz DUV modulation. As shown in fig. S15, clear switching behavior was maintained across the entire temperature range. The baseline level (DUV-Off state) varied slightly from 1.418 to 1.377 V, while the output contrast (Δsignal) remained in the range of 72.07 to 77.34 mV, with an enhanced sensitivity of 104.6 mV at 100°C. Because the PET-based platform imposes practical limits on the maximum test temperature, we additionally evaluated a photodetector fabricated on a quartz substrate and extended the temperature test range up to 200°C under the same DUV modulation condition, where stable switching behavior was also preserved (fig. S16). Next, humidity-dependent measurements were conducted by varying the relative humidity (RH) from 20 to 80%. As shown in fig. S17, the baseline signal and output contrast remained nearly constant across the full RH range, with standard deviations of 2.31 and 1.27 mV, respectively. We then investigated the dependence on ambient composition by adjusting the oxygen concentration. Stable operation was preserved when the oxygen concentration was varied from 21 to 8%, with standard deviations of 0.62 mV (baseline) and 1.21 mV (output contrast) (fig. S18). Last, pressure-dependent measurements were performed over a chamber-pressure range of 1 to 0.94 MPa. As shown in fig. S19, the device response remained stable with standard deviations of 0.66 mV (baseline) and 1.56 mV (output contrast). These results demonstrate that the device maintains reliable DUV sensing performance under practical variations in temperature, humidity, oxygen concentration, and pressure.

With the integrated, battery-powered sensor module in hand, we carried out real-time flame detection experiments to validate its functionality in relevant scenarios. We selected three different flame sources (photographs in Fig. 3C) to represent a range of fire types: (i) a butane blowtorch, (ii) a burning ethanol-based solid-fuel block, and (iii) a natural gas kitchen stove burner. The sensor device was positioned at a fixed distance of 40 cm from each flame source, and the DUV photodetector’s output was monitored while the flames were ignited and sustained. The sensor response was wirelessly transmitted and recorded in real time using a custom-made mobile application. Figure 3 (D to F) shows the recorded sensor signal as a function of time for each flame. In all cases, the sensor exhibited a clear response upon flame ignition. Starting from a baseline, the introduction of a flame produced a signal increase, confirming the system’s capability for real-time flame detection. No false alarms (such as sudden signal increases or drops) were triggered by indoor light illumination. Once the flame was extinguished or removed, the sensor signal returned to the baseline. The magnitude and shape of the sensor signals varied with the flame source, reflecting the different DUV emission characteristics of each flame.

The butane torch (Fig. 3D) exhibited the lowest responsiveness, with the maximum output signal changing by ∼23% relative to the baseline. The ethanol-based solid fuel (Fig. 3E) produced moderate responsiveness, corresponding to a maximum signal change of 59% from the baseline. The temporal profile displayed a smooth transition, consistent with the laminar and steady combustion behavior of the solid fuel. In contrast, the gas-stove flame (Fig. 3F) yielded the highest responsiveness, with the output deviating by nearly 97.8% from the baseline. Despite the differences in magnitude, all flame types were easily discernible from the baseline and from each other by the output signal of the sensor. Beyond these flame type–dependent responses, we further validated the sensor behavior under realistic scenarios. Movie S2, together with the corresponding transient plot in fig. S20, demonstrates that the output signal can be tracked continuously during dynamic flame events, confirming the feasibility of real-time monitoring in practical conditions. In addition, the dependence of the response on the flame-to-sensor distance was evaluated using a gas-stove flame (fig. S21). As the distance increased from 20 to 40 cm, the amplitude of the signal change decreased from 87.15 to 38.25%, consistent with the attenuation of the DUV intensity over distance (fig. S13). This distance-resolved sensitivity highlights the ability of the platform not only to detect the presence of a flame but also to infer its relative proximity. These tests confirm that our integrated DUV photodetector system can not only detect the presence of a flame but also capture distinguishing features of the flame source and intensity in its real-time signal.

To further contextualize these results for practical operation, we further extended the distance test and evaluated flame detection performance over a wider distance and incident-angle space (fig. S22). Specifically, a gas-stove flame was used as a representative source, and the photodetector system was positioned at incremental standoff distances from 20 to 100 cm while varying the incident angle from 30° to 150° (fig. S22A). The resulting response map (fig. S22B) and distance-dependent output trends (fig. S22C) show that the signal amplitude decreases monotonically with increasing distance, consistent with reduced incident emission intensity, and also depends on the incident angle under practical mounting geometries. As expected, the flame-induced signal becomes less pronounced at longer distances, yet a measurable output change and characteristic temporal response were still observed across the tested distance and angle conditions (fig. S22D). These results indicate that the platform provides a practical sensitivity margin for proximity flame monitoring, and the use of larger flame sources is expected to yield higher emission levels, which could further improve the signal-to-background ratio and increase the detection margin. To verify stable detection under sustained flame exposure, we performed an additional long-duration measurement using the same gas-stove flame source at a fixed distance of 40 cm and continuously monitored the output for 30 min (fig. S23). A clear transition from the flame-off baseline to the flame-on state was observed upon ignition, followed by a stable output level that remained trackable throughout the 30-min interval with only minor fluctuations.

**Fig. 4.**
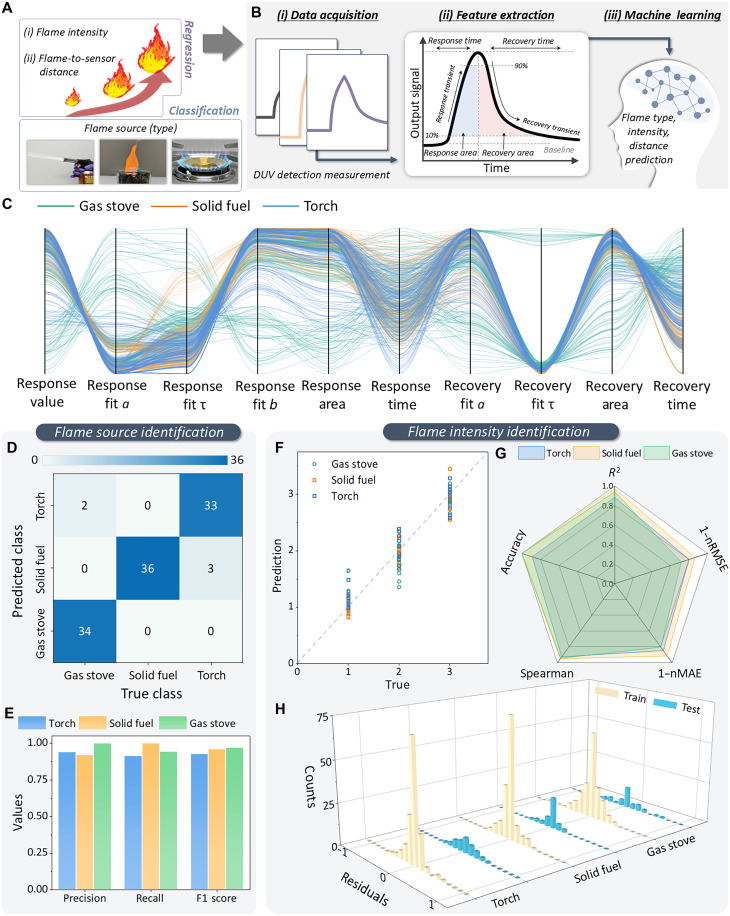
Extension of the sensing functionality by using ML. Schematic illustration of the (**A**) ML-based approach for flame identification and (**B**) sensor response feature extraction. (**C**) Extracted features according to the data recorded under different flame sources. (**D**) Confusion matrix of flame source classification results for three flame types (torch, solid fuel, and gas stove). (**E**) Comparison of precision, recall, and F1 score for each flame source. Regression analysis for flame intensity estimation showing (**F**) predicted versus true values and (**G**) radar plots of performance metrics. (**H**) Residual distribution histograms of the regression model for training and test datasets.

While the raw sensor output alone enables binary fire detection, we leveraged the rich temporal features embedded in the DUV response curves and applied ML techniques for flame source classification, intensity estimation, and distance regression (Fig. 4A). As illustrated in Fig. 4B, the ML framework consists of three stages: (i) data acquisition, where the DUV sensor records the response signals from different flame sources under varying experimental conditions; (ii) feature extraction, where representative quantitative descriptors are derived from the sensor response signals for subsequent analysis; and (iii) ML process, where these features are used in classification and regression models. The system uses classification to identify flame sources, regression to estimate flame intensity, and a second regression model to infer the distance between the flame source and the sensor. The comprehensive dataset, including variations in flame intensities and distances, enables the system to operate effectively across various real-world scenarios. For dataset organization, we conducted an expanded set of experiments to build a comprehensive and structured dataset. Sensor responses were recorded for each of the three flame types (torch, ethanol fuel, and gas stove) under systemically varied conditions. These conditions included different flame intensities (e.g., adjusting the torch gas flow rate or using larger versus smaller fuel blocks) and a range of flame-to-sensor distances (5, 10, 15, and 20 cm). Each sensor measurement produced a time-series photocurrent profile spanning the ignition, steady burning, and extinction stages of the flame. From these temporal response curves, we derived a structured set of quantitative descriptors that capture the essential dynamics of the signal. The feature set included the following: (i) temporal parameters extracted from exponential fitting of the response and recovery transients, (ii) amplitude-related metrics such as peak photocurrent and on/off ratio, and (iii) integrated measures obtained from the area under the response and recovery curves, corresponding to the accumulated DUV exposure. The exponential fitting of the sensor’s response was performed using the functional form

$$R\left(t\right)=a\cdot e^{\left(-\frac{t}{\tau }\right)}+b$$ (3)

where *a* is the amplitude, τ represents the characteristic time constant, and *b* corresponds to the steady state offset. For the recovery process, *b* was set to zero to reflect the full return to the baseline. From this fitting, 10 parameters were extracted: *a*, τ, and *b* for both response and recovery segments, the integrated areas of the response and recovery curves, and the response time corresponding to 90% rise and 10% decay of signal. All fitted lines of the sensor responses are summarized in figs. S24 to S26. The exponential fitting of *R*(*t*) provides a direct physical linkage between the sensor response and its underlying carrier trapping-detrapping kinetics. By reducing the transient behavior to a minimal set of time constants and amplitudes, this approach yields intrinsic descriptors that preserve the mechanism-specific dynamics while offering a stable and interpretable feature space for subsequent ML analysis. To investigate the distribution and latent structure of the extracted features before model training, a parallel coordinates plot was generated (Fig. 4C). Although partial overlap is observed across different flame sources, subtle yet discernible trends appear along several axes, particularly those associated with exponential-fitting parameters and integrated response values. In addition, the feature space exhibits complex multidimensional correlations between parameters, reflecting the intertwined nature of the underlying flame-sensor interactions. Such relationships are not easily captured by simple linear or threshold-based approaches, underscoring the need of ML-based modeling to effectively learn and exploit these latent dependencies. The results reveal distinct correlation patterns depending on the flame type, suggesting that flame-dependent response dynamics are embedded in the feature space. For the ML training, the dataset was randomly divided into training and test subsets, with the latter comprising 20% of the total samples. A fivefold cross-validation strategy was adopted for model training and hyperparameter optimization to ensure statistical robustness. For flame source classification and flame intensity and distance estimation, multilayer perceptron (MLP) was used. As shown in Fig. 4D, the model achieves perfect classification for solid-fuel flames and near-perfect classification for gas-stove flames. Most misclassifications are associated with the torch class, which is occasionally predicted as solid fuel. This behavior can be attributed to partial overlap in the temporal and amplitude-related response characteristics between torch and solid-fuel flames, particularly under lower intensity torch conditions. Figure 4E further presents that the model maintains high precision, recall, and F1 scores (>0.92) for gas-stove and solid-fuel flames, while the torch class exhibits slightly lower values because of its more complex temporal response dynamics. These results highlight the strength of the ML framework in disentangling the complex, intertwined feature space revealed by the parallel coordinates plot. Although the raw feature distributions exhibited substantial overlap and multidimensional correlations, the trained model successfully captured these latent relationships and translated them into accurate class boundaries, particularly for gas-stove and solid-fuel flames.

To investigate regression-based flame intensity estimation, an MLP model was used using the same framework as for classification. The predicted intensity values closely follow the ideal 1:1 relationship with the ground truth across all flame sources (Fig. 4F and figs. S27 and S28), confirming that the extracted temporal and amplitude-related descriptors provide strong predictive power. Overall performance metrics (Fig. 4G) indicate a mean coefficient of determination (*R*^2^; variance explained by the model) of 0.93, a mean accuracy (fraction of predictions within the predefined tolerance) of 0.96, and a mean Spearman correlation coefficient (rank-order agreement between predicted and true intensities) of 0.94. For unified visualization, the error-based metrics, namely the root mean square error (RMSE; squared deviation) and the mean absolute error (MAE; absolute deviation), are normalized and plotted as 1 − nRMSE and 1 − nMAE, corresponding to raw mean RMSE and mean MAE values of 0.21 and 0.15, respectively. These results demonstrate the model’s robustness in handling variations in flame intensity across different combustion sources. Figure 4H presents the residual (predicted value-true value) distributions for both training and test datasets, showing narrow peaks centered around zero with minimal spread. This indicates that the model exhibits a low systematic error and appropriate generalization across unseen data. The regression performance remains stable across torch, solid-fuel, and gas-stove sources, underscoring the model’s ability to reliably translate sensor features into accurate intensity predictions despite inherent source variability.

In addition to flame intensity estimation, we further examined the feasibility of predicting the flame-to-sensor distance using the same set of temporal and amplitude-related features. In the distance-estimation experiments, the target distance was varied from 5 to 20 cm (total range, 15 cm). The MLP regression model demonstrated strong linear agreement between predicted and actual distances across all flame sources, with coefficients of determination (*R*^2^) ranging from 0.943 to 0.991 and consistently low prediction errors, as reflected by narrow residual distributions (figs. S27 to S29). To enable a direct comparison between error- and correlation-based performance metrics on a unified 0-1 scale, the RMSE and MAE were normalized by the target distance range (5 to 20 cm). The normalized error was therefore defined as error/15, and the inverted normalized metrics (1 − nRMSE and 1 − nMAE) were used for visualization. Using this representation, 1 − nRMSE and 1 − nMAE remained consistently high across flame sources (1 − nRMSE = 0.888 to 0.965 and 1 − nMAE = 0.941 to 0.976), indicating a low absolute prediction error relative to the target range. In addition, the Spearman rank correlation coefficients were 0.962 to 0.964, supporting strong monotonic agreement between predicted and actual distances across flame types. This representation reveals consistently high regression performance across torch, solid-fuel, and gas-stove flames (fig. S30), demonstrating that the proposed platform can robustly estimate flame proximity in addition to identifying flame type and intensity within a single, unified framework. The residual distributions for both the training and test datasets were narrow and centered near zero, suggesting a low systematic error and strong generalization across flame types (fig. S31).

We further examined an overlapping-flame scenario to evaluate how the trained classifier behaves when two flame sources are simultaneously active (fig. S32A). In this additional test, a gas-stove flame and a torch flame were ignited at the same time and measured under five mixed distance conditions labeled as (Gas stove/Torch) = (20/10), (15/10), (10/10), (10/15), and (10/20) cm. Using the same trained MLP model, the predictions tended to follow the dominant contributor in the mixed signal. When the gas-stove contribution was higher (20/10 and 15/10), the gas-stove class was predicted in 60 and 50% of trials, respectively, whereas torch-dominant cases (10/10, 10/15, and 10/20) were classified as torch in 80, 90, and 90% of trials, respectively (fig. S32, B and C). These results suggest that under the controlled overlap conditions examined here, the model generally predicts the source that contributes more strongly to the measured response. A consolidated quantitative comparison of the key performance metrics of this work with prior UV-based flame sensors is summarized in table S2.

## DISCUSSION

In summary, this work presents an outdoor-compatible flame sensing platform that integrates a solar-blind DUV photodetector with flexible circuitry and data-driven analysis. Beyond demonstrating a DUV-responsive material or a standalone detector, our contribution lies in establishing a distributed, low-power, flexible sensing-node paradigm motivated by early fire detection in open environments where continuous monitoring and human access are limited. The ZTO nanocomposite photodetector provides selective DUV detection with a responsivity of 8.58 mA W^−1^ at 250 nm, subsecond response times, and stable operation under mechanical deformation and long-term storage. The device also exhibited strong solar-blind selectivity with a DUV/visible rejection ratio of 5075. By directly integrating this photodetector with flexible circuitry and wireless communication, we implemented a unit that can be deployed in the field like an “electronic sticker” on trees or structures. This compact unit is suitable for attachment to low-height vegetation or fire-prone infrastructure, enabling proximity monitoring near likely ignition locations. The device maintained high performance under bending stress, addressing the challenge of developing flexible flame sensors for complex environments. In addition, the platform preserved stable sensing performance under practical variations in temperature, humidity, oxygen concentration, and pressure. Last, through ML, we extended the device’s functionality from simple detection to an information-rich analysis, capable of predicting flame source, intensity, and relative distance, with coefficients of determination ranging from 0.94 to 0.99 depending on the task and flame type. This combination of capabilities is unprecedented in the wildfire sensing arena and could enhance early warning systems.

Looking ahead, the platform can be further strengthened for real-world adoption in several directions. On the algorithmic side, the ML framework could be improved by expanding the training dataset to include a wider range of flame sources and more complex scenarios and by refining waveform-derived feature representations to enhance generalization. In addition, for very long flame-on periods where the signal may gradually drift, incorporating drift-robust decision rules, such as slope-based criteria, could be explored to improve reliability in field operation. From a practical installation perspective, conformal field mounting on irregular surfaces could be facilitated by exploring eco-friendly adhesive interlayers, such as polydopamine-based coatings on the backside of the substrate. In addition, end-of-life considerations could be addressed by exploring more environmentally benign or transient material sets for the substrate, interconnects, packaging, and the power module, enabling retrieval-friendly or potentially biodegradable implementations for unattended outdoor settings. Last, response dynamics and overall system performance could be further improved through continued reduction of trap-related effects via film and interface optimization, together with readout time-constant optimization.

## MATERIALS AND METHODS

### Material preparation, NP synthesis, and device fabrication

#### Reagents

Acetylacetone (≥99%), chloroform (≥99.8%), dimethyl sulfoxide (≥99.9%), zinc acetate dihydrate (ACS reagent, ≥98%), zinc nitrate hexahydrate (98%), tin(IV) chloride pentahydrate (98%), 2-methoxyethanol (99.8%), copper(I) thiocyanate (99%), and PEDOT:PSS (3.0 to 4.0% in H_2_O, high conductive grade) were purchased from Sigma-Aldrich (US). Ethanol (anhydrous, 94 to 96%, EtOH) was supplied by Alfa Aesar (US). The ammonia solution (25%) was purchased from Supelco (US). The tin(II) chloride dihydrate was purchased from Samchun (South Korea).

#### Synthesis of ZTO NPs and precursor solution preparation

ZTO NPs were synthesized via a hydrothermal method. Zinc acetate dihydrate (6 mmol) and tin(IV) chloride pentahydrate (12 mmol) were dissolved in 160 ml of an aqueous NaOH solution (0.2 M) under magnetic stirring for 1 hour. The mixed precursor solution was subsequently transferred into a 200-ml Teflon-lined autoclave and heated at 160°C for 12 hours. After naturally cooling to room temperature, the precipitated products were collected by centrifugation and washed repeatedly with ethanol and deionized water to remove residuals. The purified ZTO NPs were dried under ambient conditions for 12 hours and subsequently calcined at 500°C for 3 hours in an air atmosphere. The sol-gel–based zinc and tin precursor was prepared as follows. For the zinc precursor (vial 1), 1.190 g of zinc nitrate hexahydrate was dissolved in 20 ml of 2-methoxyethanol, followed by the addition of 0.8 ml of acetylacetone. The mixture was stirred for 20 min, after which 456 μl of 25% ammonia solution was added, and the solution was further stirred for 1 hour. For the tin precursor (vial 2), 758.4 mg of tin(II) chloride dihydrate was dissolved in 20 ml of 2-methoxyethanol with 0.8 ml of acetylacetone. After stirring for 20 min, 228 μl of 25% ammonia solution was added, and the mixture was stirred for 1 hour. Subsequently, equal volumes (10 ml each) of the zinc and tin precursor solutions were combined (vial 3) and stirred for 1 hour to obtain the sol-gel–based zinc tin precursor. The NP-based zinc tin precursor (vial 4) was prepared by mixing 7.5 ml of chloroform and 2.5 ml of ethanol, followed by the addition of 100 mg of hydrothermally synthesized ZTO NPs. The dispersion was stirred for 24 hour. Last, equal volumes (5 ml each) of the sol-gel precursor solution (vial 3) and the NP precursor solution (vial 4) were mixed to yield 10 ml of the ZTO NPs@sol-gel solution. The CuSCN solution (10 mg/ml) was prepared by dissolving 100 mg of copper(I) thiocyanate in 10 ml of dimethyl sulfoxide. The mixture was sonicated at room temperature for 24 hour until it was completely dissolved. The PEDOT:PSS solution was prepared by mixing 7.5 ml of PEDOT:PSS with 2.5 ml of isopropyl alcohol. The mixture was stirred with a magnetic bar for 24 hours at room temperature until a uniform solution was formed.

#### Device fabrication

The ITO/PET substrate and FPCB were fixed onto a glass slide (76 by 26 mm) using Kapton tape. The substrate was exposed to UV-ozone for 15 min to render the surface hydrophilic. Subsequently, 200 μl of the ZTO NPs@sol-gel solution was drop-cast onto the surface and spin-coated at 1500 rpm for 30 s. The coated substrate was then exposed to UV-ozone for an additional 15 min, followed by thermal annealing at 120°C for 30 min on a hot plate. Before deposition of the CuSCN layer, the substrate was again subjected to UV-ozone treatment for 15 min. The CuSCN solution (200 μl) was drop-cast and spin-coated at 1500 rpm for 30 s, followed immediately by thermal annealing at 100°C for 15 min. Last, the prepared PEDOT:PSS solution (sonicated at room temperature for 1 hour before use) was deposited by drop-casting 200 μl of the solution and spin-coating at 2400 rpm for 30 s. The film was subsequently dried on a hot plate at 100°C for 1 hour. For reliable electrical connection during measurements, a thin Ag paste layer was applied on the predefined electrode contact pads as an assistive contact layer.

### Characterization of the materials and devices

#### Material characterization

The morphology and microstructure of the ZTO NPs and nanocomposite films were characterized using a field-emission scanning electron microscope (SU8600, Hitachi) equipped with an energy-dispersive spectrometer (SU8600, Hitachi). The crystalline structure of the synthesized ZTO NPs was examined by x-ray diffraction (Smartlab, Rigaku). Absorption spectra were characterized through a UV/visible spectrometer (LAMDA 750, PerkinElmer), and the optical bandgaps were estimated from Tauc plots derived from the absorption data. Photoluminescence and time-resolved photoluminescence measurements were conducted using a spectroscopic system (Fluorolog3, HORIBA) equipped with a pulsed excitation source.

#### Device characterization

The optoelectronic characteristics of the DUV photodetectors were evaluated using a semiconductor parameter analyzer (Keithley 4200A-SCS, Tektronix) and a probe station. A DUV light source with a peak wavelength of 265 nm (M265L5, Thorlabs Inc.) was used to illuminate the devices. The photoresponse, current-voltage characteristics, and transient response were recorded under controlled illumination conditions. Ambient-light measurements were performed under laboratory white-LED illumination (microscope-mounted LED). The ambient light source’s broadband irradiance was monitored as 271 W m^−2^ using a Si photodiode–based radiometer (Daystar DS-05A; nominal spectral response of 0.3 to 1.1 μm). For standardized strong-illumination testing, AM1.5G solar-simulator measurements were conducted at 1 sun (100 mW cm^−2^ total irradiance). Spectral responsivity measurements were performed using a custom-built system equipped with a mercury-xenon lamp as the broadband light source and wavelength-selective optical filters (K3100 sensor measurement system, McScience Inc.). Mechanical bending tests were performed using a custom-built bending setup, where the devices were subjected to repeated bending cycles with defined radii while monitoring the photocurrent response. For the 180-day storage test, the device was stored in a laboratory environment at room temperature (25°C) under dark conditions (in a closed container) and ambient humidity (20 to 30% RH) without encapsulation. After storage, the device was remeasured under the same electrical and optical test conditions as the initial characterization.

### Hardware and software construction

#### Embedded system architecture

The wireless flame detection device was monolithically integrated onto a transparent PET-based FPCB. The overall dimensions of the board were 64.8 mm by 16.9 mm by 0.1 mm. Device power was provided by a compact, rechargeable lithium-ion battery (3.7 V, 12 mA·hour, GPE) featuring a flexible and thin structure. A low-dropout regulator (TPS7A0333PDQNR, Texas Instruments) was used to convert the battery input voltage into a stable 3.3-V dc supply for the entire sensor circuit. The photocurrent generated by the DUV sensor was converted and amplified into a voltage signal via a current-to-voltage converter based on an operational amplifier (TLV8802, Texas Instruments), a feedback resistor (50 MΩ, Stackpole Electronics), and a capacitor (100 pF, YAGEO). The amplified voltage signal was digitized by the analog-to-digital converter embedded in a BLE SoC (ISP1807, Insight SIP), which also handled wireless communication and digital signal processing of analog sensor outputs. To enable low-power operation, the SoC was programmed to remain inactive during idle states and to activate only during sensing or transmission periods. The digitized signal was wirelessly transmitted in real time via the BLE SoC’s integrated antenna to an external mobile device. This device was designed to intuitively visualize the received DUV signals and included a data logging function for continuous monitoring and storage.

#### Feature extraction, data processing, and ML model training

Photocurrent response signals were collected from three flame sources (torch, ethanol solid fuel, and gas stove) at four flame-to-sensor distances (5, 10, 15, and 20 cm) and three intensity levels per source, yielding a total of 540 measurements. Each measurement consisted of a time-resolved photocurrent profile spanning the ignition, steady burning, and extinction stages. To parameterize the temporal response, exponential fitting was applied to both the rise and recovery transients, and 10 quantitative descriptors were extracted from each curve, including amplitude and time constant–related parameters, integrated response and recovery areas, and characteristic response/recovery times. These descriptors provide a compact and physically meaningful representation of the DUV photocurrent dynamics. Data preprocessing and feature engineering were carried out in Python using NumPy, Pandas, and SciPy for numerical analysis and curve fitting. Scikit-learn was used for model development, including feature scaling, training, and performance evaluation. The dataset was randomly partitioned into training (80%) and test (20%) subsets, and a fivefold cross-validation strategy was used for model training and hyperparameter optimization. Hyperparameter tuning was conducted using GridSearchCV and RandomizedSearchCV, with search spaces tailored to each algorithm. The neural network model (MLP) was tuned for hidden layer size, activation functions, and solver parameters. Model performance was evaluated using accuracy, precision, recall, and F1 score for classification and coefficient of determination (*R*^2^), RMSE, MAE, and Spearman correlation for regression. Residual analysis was further performed to assess generalization and systematic bias in model predictions. The detail codes are represented in Supplementary Text S1.

## Supplementary Materials

### The PDF file includes:

Supplementary Text S1 Tables S1 and S2 Figs. S1 to S32 Legends for movies S1 and S2 References
