# Supplementary material for: An integrated wireless deep-UV sensing system for intelligent early fire detection
Source: Sci Adv. 2026 May 27;12(22):eaef4143. doi: 10.1126/sciadv.aef4143 (PMC13215196; doi:10.1126/sciadv.aef4143)
Supplement: Supplementary file 1 — Supplementary Text S1 Tables S1 and S2 Figs. S1 to S32 Legends for movies S1 and S2 References [file sciadv.aef4143_sm.pdf]

Science Advances
AAAS

Supplementary Materials for

An integrated wireless deep-UV sensing system for intelligent early fire detection

Taehyun Park *et al.*

Corresponding author: Jaehyun Hur, jhhur@gachon.ac.kr; Garam Bae, grbae@dankook.ac.kr; Seyong Oh, seyongoh89@hanyang.ac.kr; Hocheon Yoo, hocheon@hanyang.ac.kr

Sci. Adv. **12**, eaef4143 (2026)
DOI: 10.1126/sciadv.aef4143

The PDF file includes:

Supplementary Text S1
Tables S1 and S2
Figs. S1 to S32
Legends for movies S1 and S2
References

Other Supplementary Material for this manuscript includes the following:

Movies S1 and S2

Supplementary Text S1.

Time-Resolved Signal Segmentation and State Labeling

The time-series signal is partitioned into four distinct regimes based on its temporal evolution relative to the minimum signal point, which corresponds to the peak response. Each segment is assigned a label representing a physically meaningful state of the system:

- **Label 0 (Baseline region):** This region represents the steady-state baseline prior to stimulus exposure, where the signal remains unperturbed.
- **Label 1 (Response region):** This regime captures the active response phase, where the signal evolves toward its extremum, refle cting the system's reaction to the applied stimulus.
- **Label 2 (Recovery region):** This region corresponds to the recovery dynamics following the peak response, during which the sys tem relaxes toward its baseline state.
- **Label 3 (Post-response steady region):** This regime represents the post-recovery steady state, where the signal stabilizes after th e completion of the response cycle.

(Python code)

```
def label_signal(df, t_col, s_col):
    df = df[[t_col, s_col]].dropna().copy()
    t_min = df.loc[df[s_col].idxmin(), t_col]
    df["label"] = np.select([df[t_col] < t_min - 5, df[t_col] < t_min, df[t_col] <= t_min + 10],[0, 1, 2], default=3)
    baseline = df.loc[df["label"] == 0, s_col].mean()
    df["Response"] = (df[s_col] - baseline) / baseline * 100
    return df
```

Exponential Fitting of Response and Recovery Dynamics

The transient behavior of the sensor signal was quantitatively analyzed by fitting the response and recovery regimes using exponential models. Two functional forms were employed: an exponential decay with offset for the response phase and a standard exponential decay for the recovery phase. These models capture the asymmetric kinetics typically observed in adsorption–desorption processes and enable extraction of characteristic dynamic parameters.

For the response regime (label 1), the signal was modeled using an offset exponential function, $y(t) = A\exp(-t/\tau) + C$, where Arepresents the amplitude of the response, $\tau$is the characteristic response time constant, and Caccounts for the residual baseline offset. The time axis was shifted such that the initial response point corresponds to $t = 0$, ensuring numerical stability during fitting. Initial parameter estimates were derived directly from the data range to facilitate convergence of the nonlinear least-squares optimization.

The recovery regime (label 2) was modeled using a simpler exponential decay function, $y(t) = A\exp(-t/\tau)$, reflecting the relaxation of the system toward equilibrium without requiring an offset term. This formulation is consistent with the assumption that the signal asymptotically approaches the baseline value.

In addition to parameter estimation, the integrated response and recovery magnitudes were quantified by evaluating the definite integral of the fitted functions over the corresponding time intervals. These integrated areas provide a cumulative measure of the transient signal evolution and complement the instantaneous amplitude and time constant parameters.

To further characterize the dynamic behavior in a model-independent manner, characteristic times were extracted directly from the experimental data. The response time was defined as the time required to reach 80% of the maximum response (minimum signal value), while the recovery time was defined as the time required to recover 80% toward the baseline. These definitions enable consistent comparison across different sensor conditions and provide an intuitive metric of system kinetics independent of the fitting model.

This combined approach, incorporating both model-based (amplitude, time constant) and data-driven (characteristic time) descriptors, enables a comprehensive evaluation of the sensor's transient performance.

(Python code)

```
def exp_decay_with_offset(t, A, tau, C):
    return A * np.exp(-t / tau) + C
```

```python
def exp_decay(t, A, tau):
    return A * np.exp(-t / tau)

def fit_response(df, t_col, y_col="Response"):
    resp_df = df[df["label"] == 1].copy()
    t = resp_df[t_col].values
    y = resp_df[y_col].values
    t_shift = t - t.min()
    p0 = (y.max() - y.min(), 1, y.min())
    popt, _ = curve_fit(exp_decay_with_offset, t_shift, y, p0=p0)
    A, tau, C = popt
    response_area, _ = quad(lambda t: exp_decay_with_offset(t, *popt), 0, t_shift.max())
    response_time = t[np.argmin(np.abs(y - 0.8 * y.min()))] - t.min()
    return A, tau, C, response_area, response_time

def fit_recovery(df, t_col, y_col="Response"):
    rec_df = df[df["label"] == 2].copy()
    t = rec_df[t_col].values
    y = rec_df[y_col].values
    t_shift = t - t.min()
    p0 = (y.min(), 1)
    popt, _ = curve_fit(exp_decay, t_shift, y, p0=p0)
    A, tau = popt
    recovery_area, _ = quad(lambda t: exp_decay(t, *popt), 0, t_shift.max())
    recovery_time = t[np.argmin(np.abs(y - (y[0] + 0.8 * (0 - y[0]))))] - t.min()
    return A, tau, recovery_area, recovery_time
```

Machine Learning Model Training and Evaluation

A multilayer perceptron (MLP) model was implemented using the scikit-learn framework. Prior to training, the dataset was randomly divided into training and test subsets with a ratio of 80:20. To ensure numerical consistency across input features, standardization was applied through a pipeline combining StandardScaler and the selected model. Hyperparameter optimization was performed using GridSearchCV, with the search space defined by a parameter grid tailored to the MLP architecture and training configuration.

Model selection was carried out using 5-fold cross-validation on the training dataset. In this procedure, the training data were repeatedly partitioned into five subsets, allowing the model to be trained on four folds and validated on the remaining fold in an iterative manner. The hyperparameter combination yielding the best cross-validation performance was selected as the final estimator.

After optimization, the best-performing model was applied to the held-out test dataset to generate predictions. This design ensures that the final performance metrics are evaluated on previously unseen data, providing an assessment of the model's generalization capability.

Separate evaluation functions were used for classification and regression tasks. For classification, model performance was quantified using accuracy, precision, recall, and F1-score, all calculated with weighted averaging to account for class imbalance. For regression, the predictive performance was assessed using the coefficient of determination (R^2), root mean squared error (RMSE), and mean absolute error (MAE). In addition, residuals, defined as the difference between true and predicted values, were calculated to examine systematic deviations and prediction error patterns.

This modular implementation separates model training from performance evaluation, thereby improving code clarity, reproducibility, and flexibility for application to both classification and regression problems.

(Python code)

```python
def train_mlp(X, y, model, param_grid, random_state=42):
    X_tr, X_te, y_tr, y_te = train_test_split(X, y, test_size=0.2, random_state=random_state)
    pipe = Pipeline([("scaler", StandardScaler()), ("model", model)])
    cv = KFold(n_splits=5, shuffle=True, random_state=random_state)
    best = GridSearchCV(pipe, param_grid, cv=cv, n_jobs=-1).fit(X_tr, y_tr).best_estimator_
    y_pred = best.predict(X_te)
    return best, y_te, y_pred
```

```
def evaluate_classification(y_true, y_pred):
    return {"accuracy": accuracy_score(y_true, y_pred),
        "precision": precision_score(y_true, y_pred, average="weighted"),
        "recall": recall_score(y_true, y_pred, average="weighted"),
        "f1": f1_score(y_true, y_pred, average="weighted"),}

def evaluate_regression(y_true, y_pred):
    return {"r2": r2_score(y_true, y_pred),
    "rmse": np.sqrt(mean_squared_error(y_true, y_pred)),
    "mae": mean_absolute_error(y_true, y_pred),
    "residuals": y_true - y_pred,}
```

Table. S1.

Summary of the fitted time constants and associated parameters obtained from time-resolved photoluminescence (TRPL) decay curves.

System	Biexponential parameters			
	A_1	A_2	τ_1 (ns)	τ_2 (ns)
ZTO	41.34	4.19	24.3	386.5
ZTO/CuSCN	25.9	3.63	33.5	481.9

Table. S2.

Comparison of detection performance, maximum distance, and stability of UV-based flame sensors.

Active materials	Responsivity	Rise/decay time	Maximum flame detection distance	Rejection ratio	Long-term stabilit y	Operation stability evaluation	Band-pass filter	Flexibility	Ability for flame soure classification and intensity regression	Refs.
$MAPbI_3$ + phosphor	26.9 $mA \cdot W^{-1}$ (0 V, 265 nm)	50.16/51.99 µs (0 V, 265 nm)	N/A	N/A	≈ 92 days	Waterproof	Used	No	N/A	26
Eu–MOF	≈ 0.22 $mA \cdot W^{-1}$ (0 V, 254 nm)	98/122 ms (0 V, 254 nm)	N/A	≈ 40 (254/365 nm)	30 days	N/A	None	No	N/A	49
La_2O_3/ ε-Ga_2O_3	1.67 $mA \cdot W^{-1}$ (0 V, 254 nm)	142.9/135.8 ms (0 V, 254 nm)	5 cm	N/A	N/A	Thermal	None	No	N/A	28
ε-Ga_2O_3	20.2 $A \cdot W^{-1}$ (-10 V, 254 nm)	44/75 ms (0 V, 254 nm)	N/A	N/A	N/A	Thermal	None	No	N/A	27
$CaTiO_3$/ NiO	16.7 $mA \cdot W^{-1}$ (0 V, 254 nm)	260/72 ms (0 V, 254 nm)	60 cm	2.9×10^4 (254 nm/UV-A)	502 days	N/A	None	No	N/A	17
Ga_2O_3	3,800 $mA \cdot W^{-1}$ (N/A, 250 nm)	N/A	8 cm	≈ 10^2 (232/700 nm)	N/A	N/A	None	No	N/A	50
Cr/ZnSSe	N/A	N/A	10 cm	≈10^4 (250 nm/long-wavelength)	N/A	N/A	Used	No	N/A	51
β-Ga_2O_3	37 $mA \cdot W^{-1}$ (0 V, 250 nm)	9/9 ms (0 V)	8 cm	1.5×10^4 (250/300 nm)	N/A	N/A	None	No	N/A	52
Diamond	2 $A \cdot W^{-1}$ (N/A, 210 nm)	N/A	6 cm	N/A	N/A	N/A	None	No	N/A	53

n-$Al_{0.44}Ga_{0.56}N$/ i-$Al_{0.44}Ga_{0.56}N$/ p-GaN	N/A	N/A	N/A	N/A	N/A	N/A	Used	No	N/A	54
CuSCN/ ZTO NC	8.8 $mA \cdot W^{-1}$ (0 V, 250 nm)	71/154 ms (0 V, 265 nm)	100 cm	5,075 (265 /550 nm)	180 days	Thermal, Humidity, Pressure, Oxygen concertation	None	Yes	Classification, Regression	This work

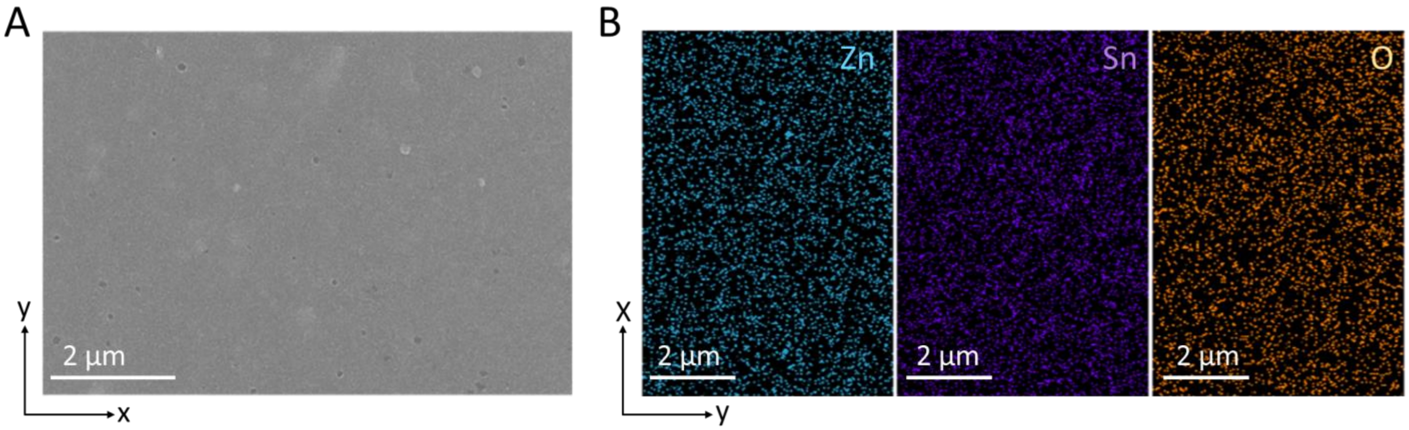

Fig. S1.

Morphology and elemental distribution of the ZTO nanocomposite film. (A) Top-view SEM image of the ZTO nanocomposite film. (B) Corresponding EDS elemental mapping images.

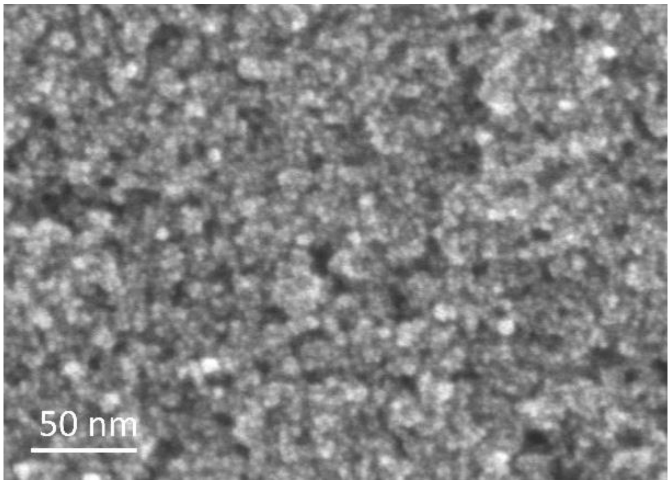

Fig. S2.

Morphology of the synthesized ZTO nanoparticles. Top-view SEM image of the ZTO NPs film.

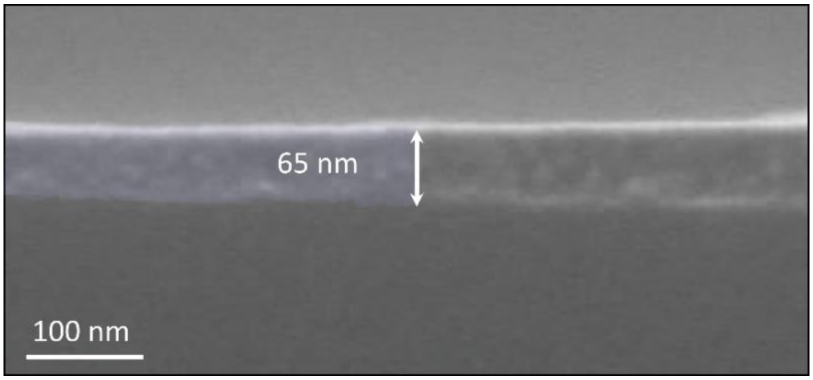

Fig. S3.

Cross-sectional structure of the composite film. Cross-sectional SEM image of the ZTO nanocomposite film.

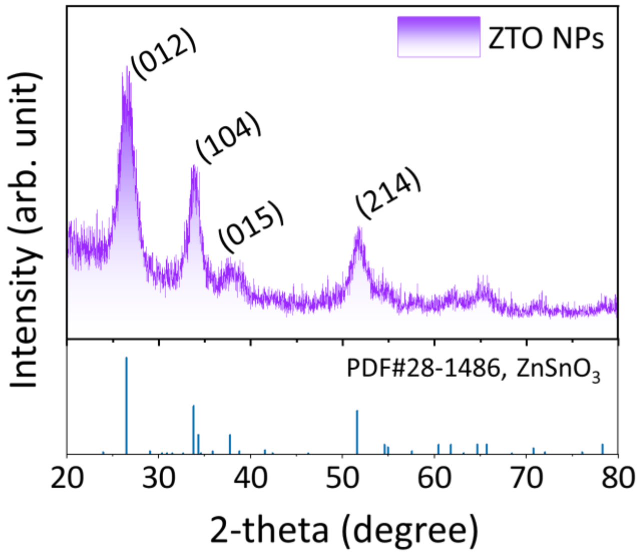

Fig. S4.

Crystallographic characteristics of the ZTO NPs. X-ray diffraction pattern of the synthesized ZTO NPs.

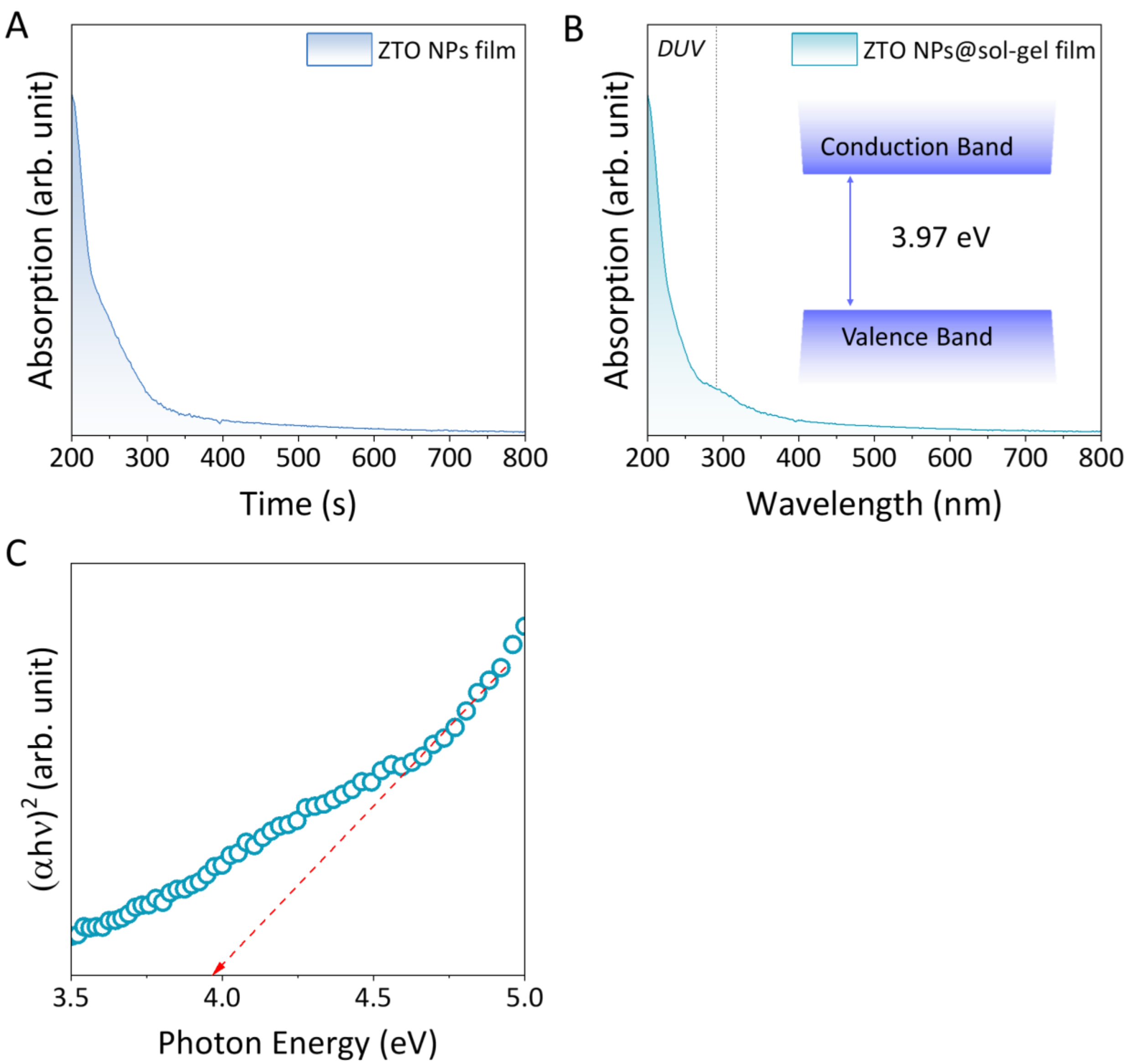

Fig. S5.

Optical absorption properties of ZTO NPs and ZTO NPs-embedded nanocomposite films. (A) UV-visible absorption spectrum of (A) ZTO NPs films, and (B) ZTO NPs-embedded nanocomposite films. (C) Tauc plot used for optical band gap estimation.

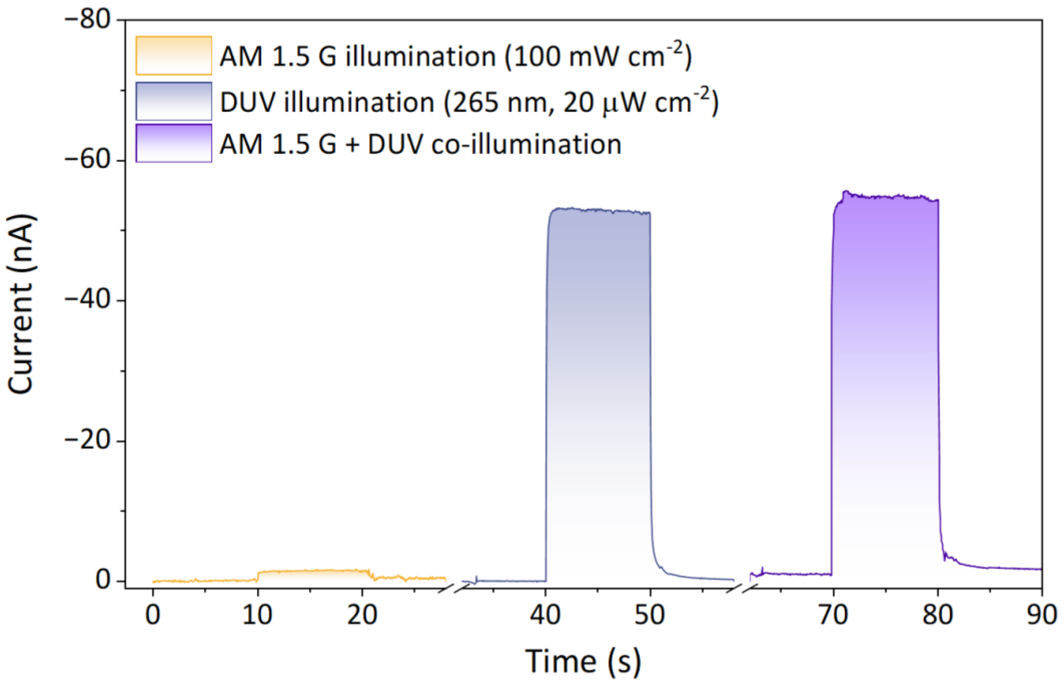

Fig. S6.

Photo response of the proposed device under AM 1.5G illumination and DUV excitation. Transient current traces measured under AM 1.5G illumination, DUV (265 nm) illumination, and AM 1.5G + DUV combined illumination, showing negligible response to AM 1.5G alone and a pronounced current change under DUV excitation.

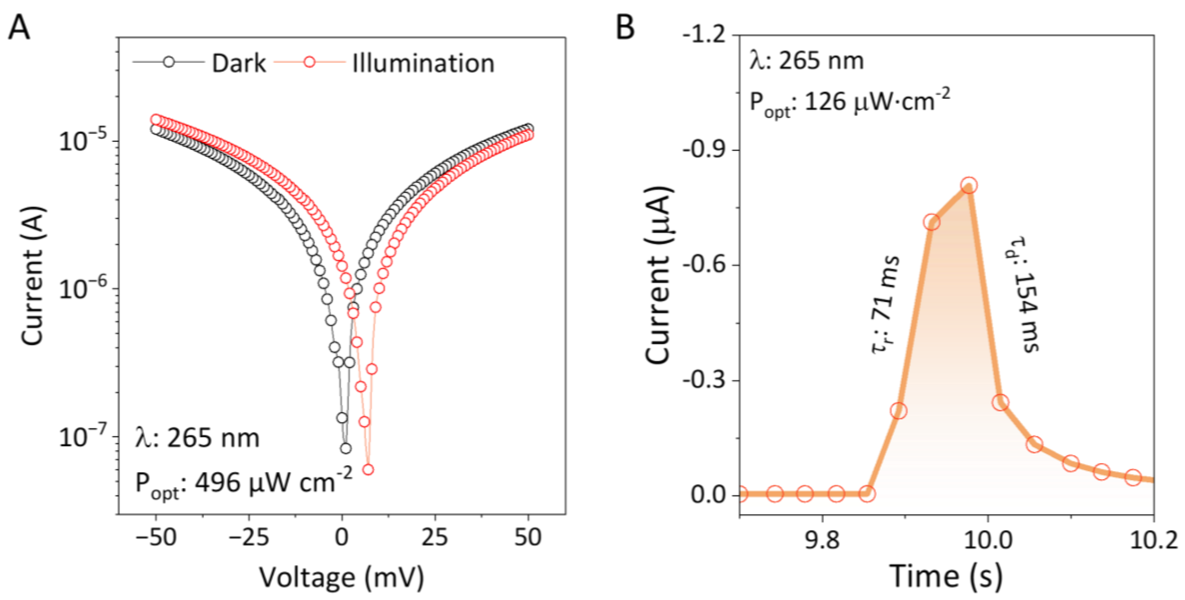

Fig. S7.

Optoelectronic characteristics of the ZTO-based DUV photodetector. (A) Current-voltage characteristics measured under dark and DUV illumination. (B) Current-time responses under the same conditions.

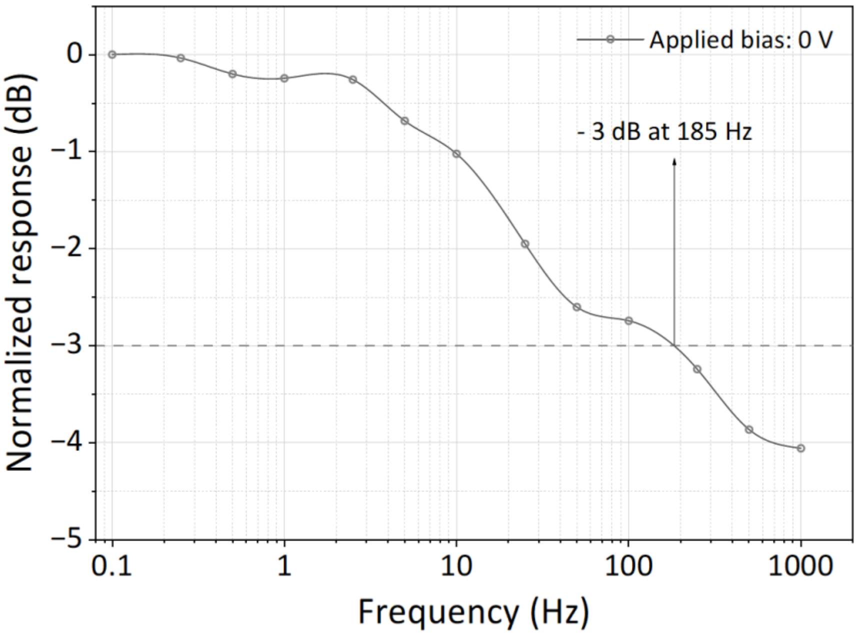

Fig. S8.

Frequency response of the proposed DUV photodetector. Normalized responsivity (output amplitude) measured as a function of DUV modulation frequency under an applied bias of 0 V.

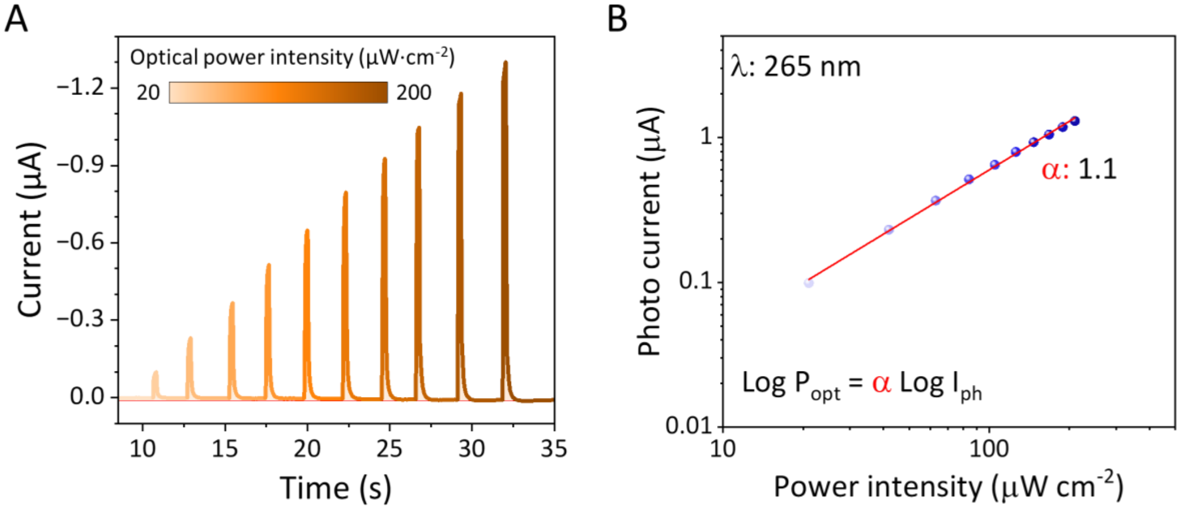

Fig. S9.

Photoresponse as a function of incident optical power.(A) Transient photocurrent responses under varying DUV power intensities. (B) Corresponding current-power plot.

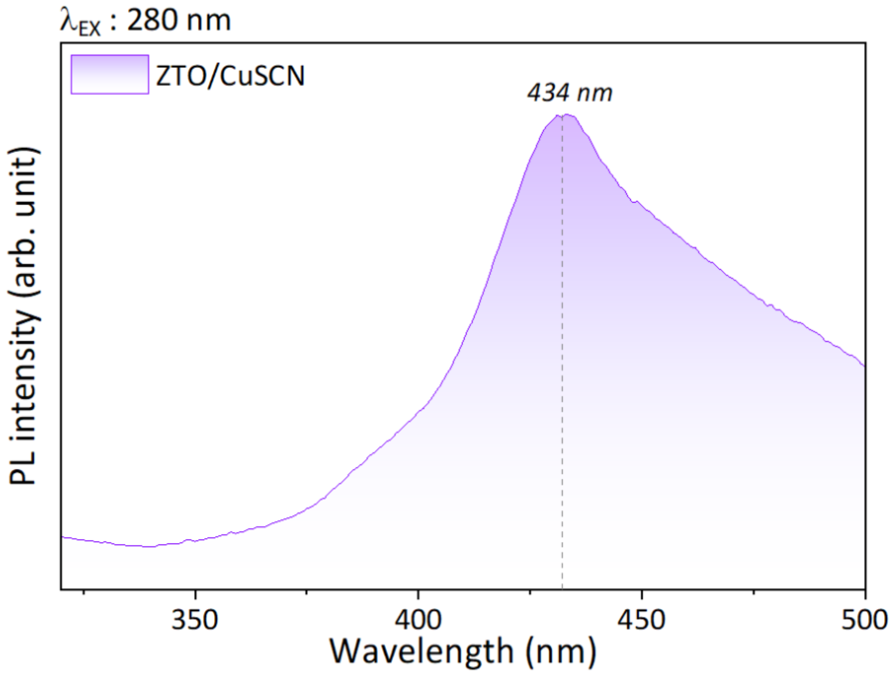

Fig. S10.

Photoluminescence spectra of the ZTO/CuSCN heterojunction film. Room-temperature photoluminescence spectra.

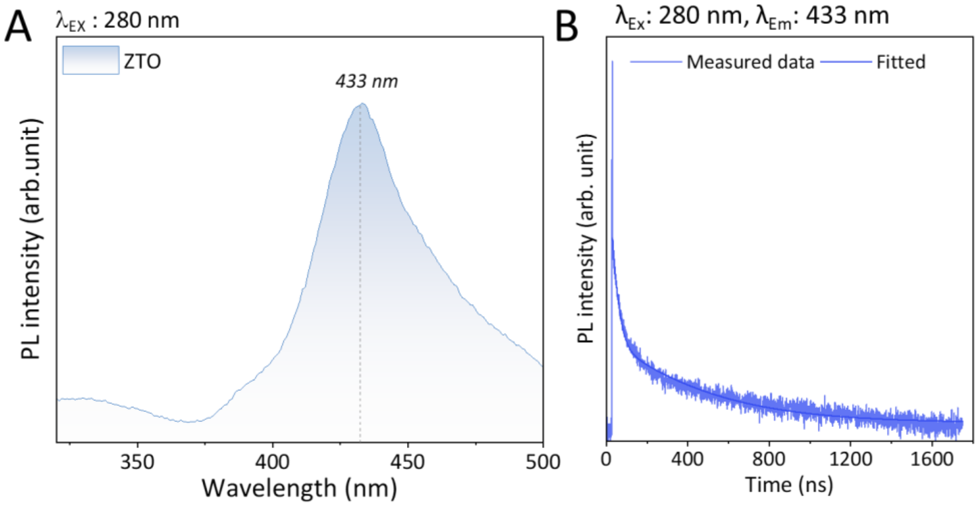

Fig. S11.

Photoluminescence and carrier dynamics of the ZTO nanocomposite film. (A) Steady-state photoluminescence spectra. (B) Time-resolved photoluminescence decay curves.

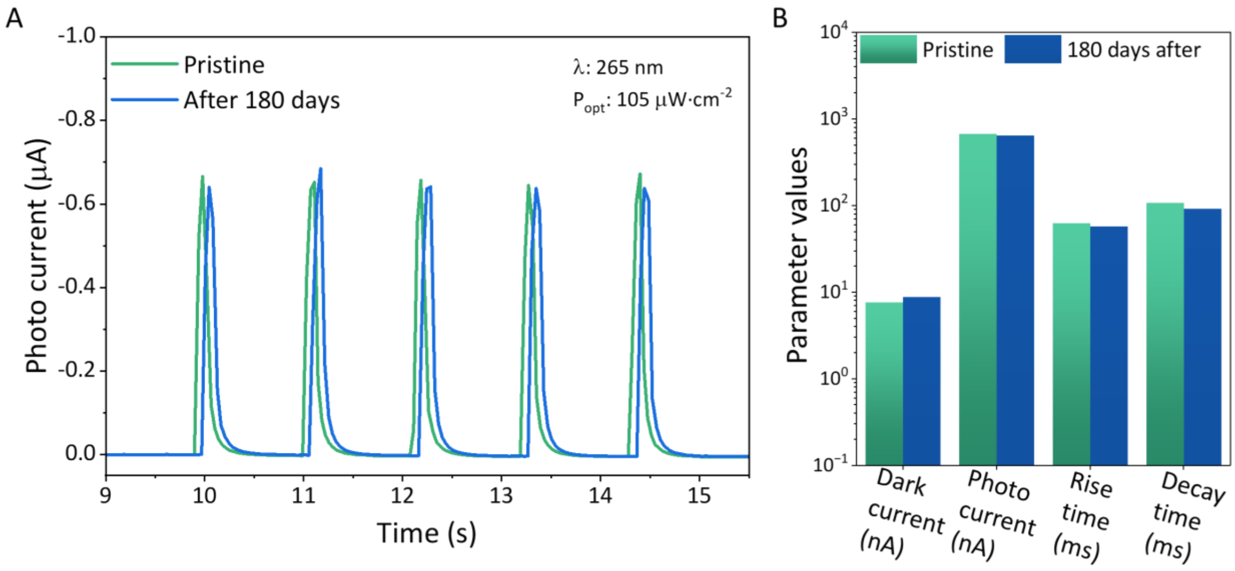

Fig. S12.

Long-term stability of the DUV photodetector under pulsed illumination. (A) Transient photoresponses recorded with five optical pulses in the pristine state and after 180 days. (B) Specific parameter values extracted from current-time characterization results.

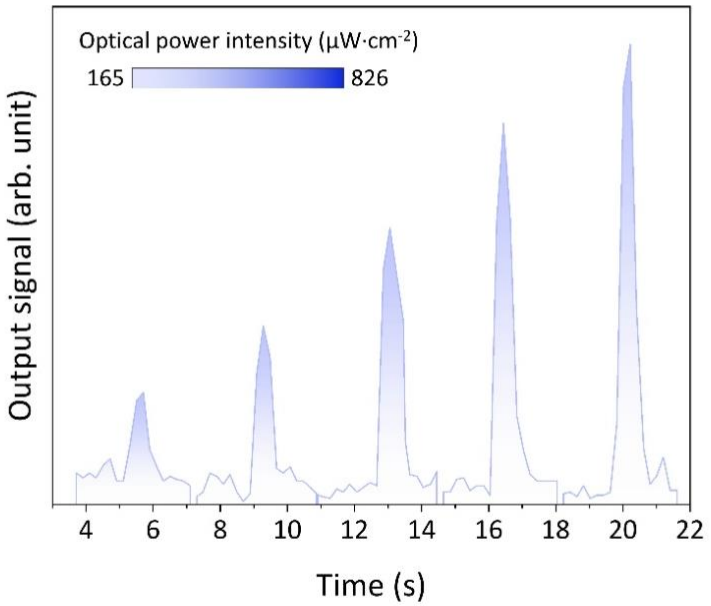

Fig. S13.

Transient photoresponses of the circuit-integrated ZTO DUV photodetector. Current-time responses measured under different 265 nm DUV optical power densities.

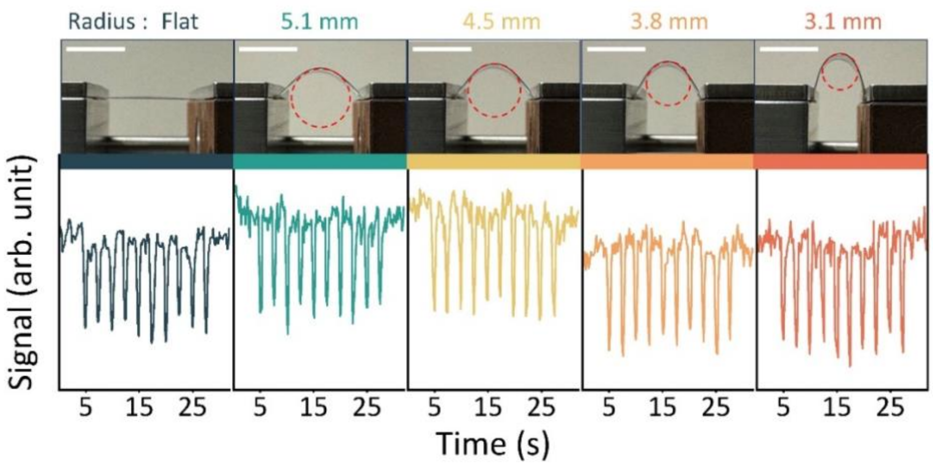

Fig. S14.

Mechanical stability of the circuit-integrated flexible flame sensor. Device operation under different bending radius. Scale bars represent 1 cm.

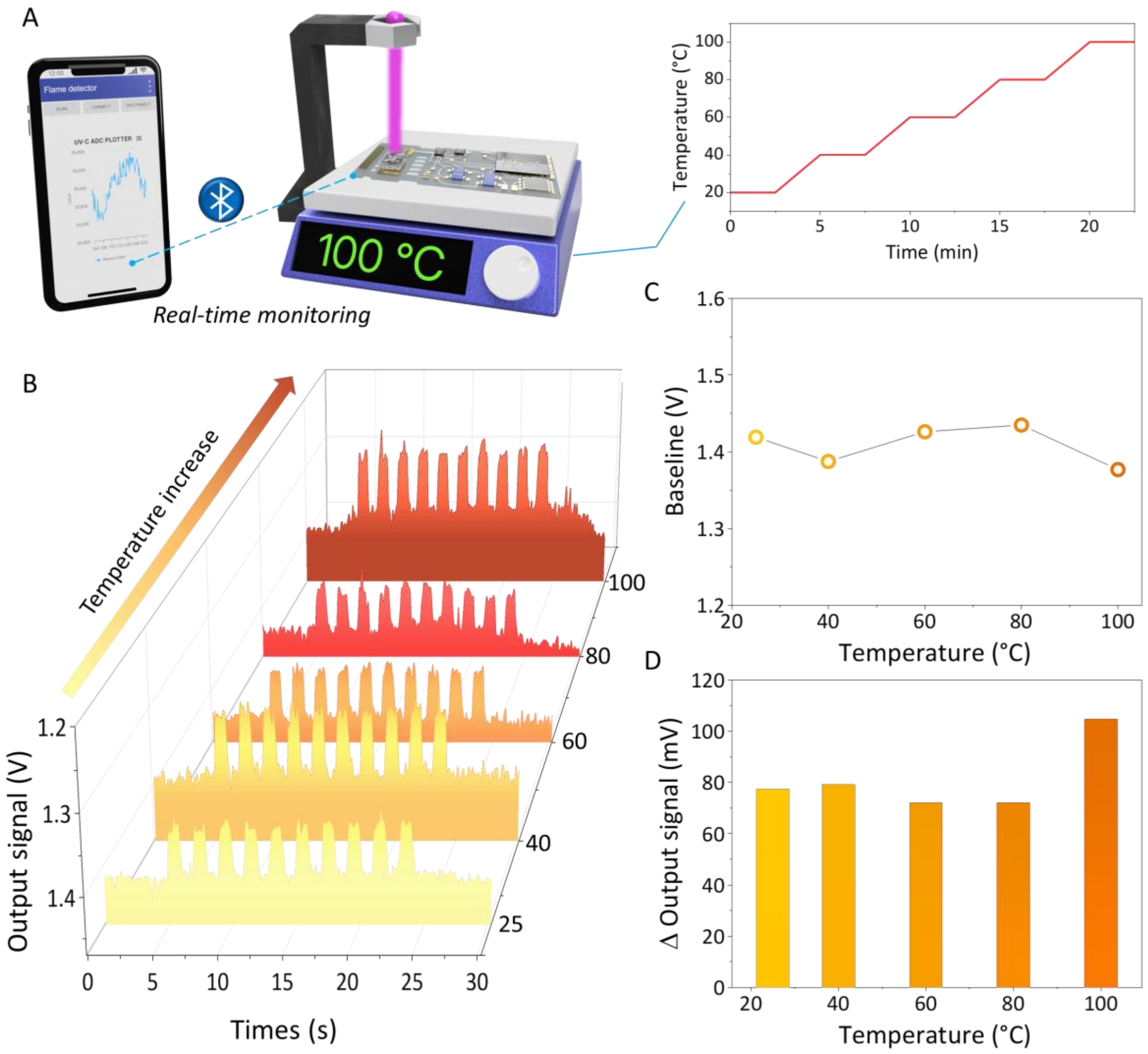

Fig. S15.

Temperature durability evaluation of the flexible sensor-board device. (A) Schematic illustration of the temperature durability test setup, where the flexible sensor-board device was placed on a temperature-controlled stage and illuminated with a DUV source. (B) Transient output traces recorded under 0.5 Hz DUV modulation at different temperatures (20 -100 °C, in 20 °C increments). (C) Baseline output level in the DUV-Off state as a function of temperature. (D) Output contrast (Δsignal) extracted from the transient response as a function of temperature.

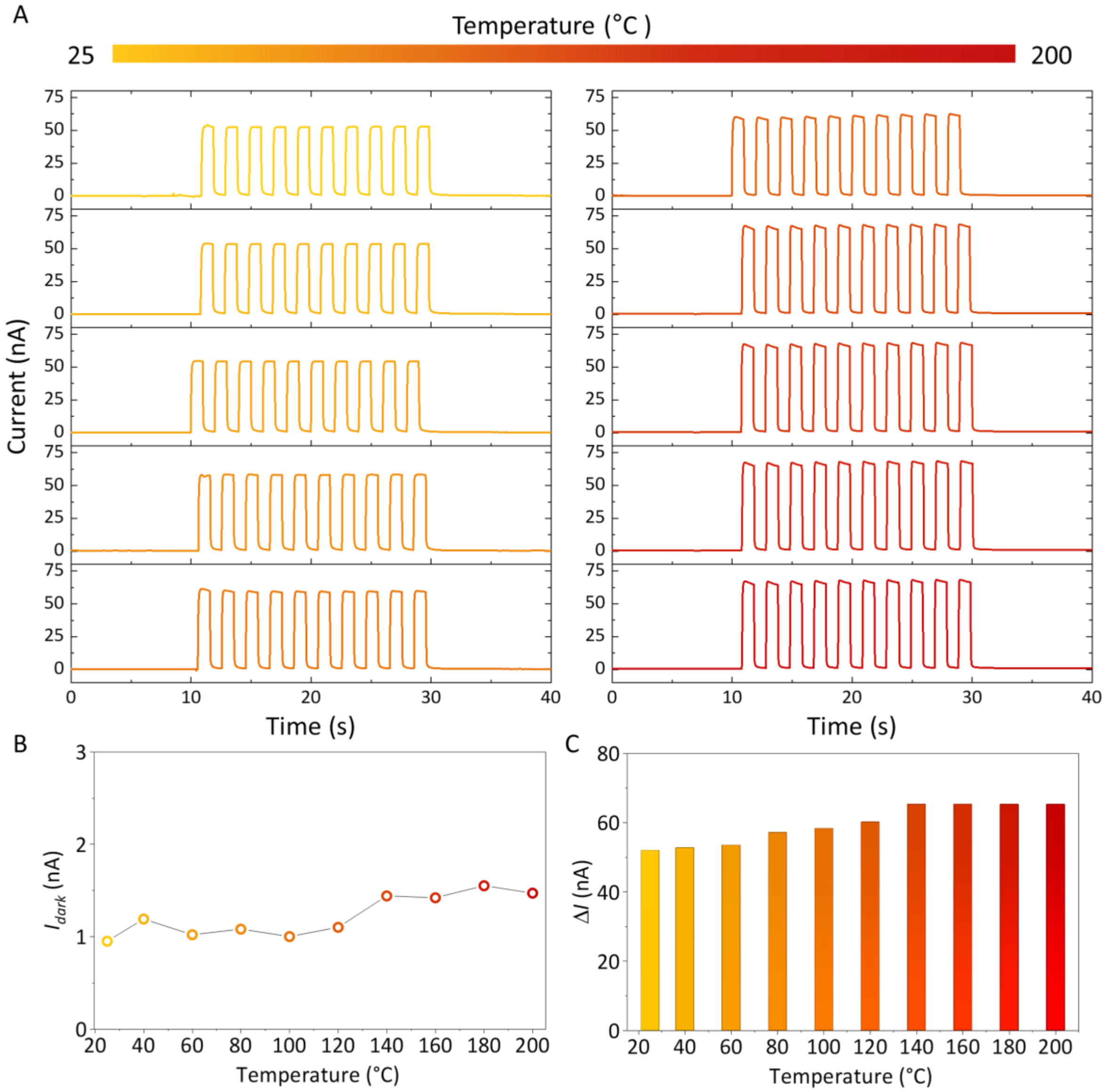

Fig. S16.

Temperature durability evaluation of the quartz-based DUV photodetector. (A) Transient photocurrent traces recorded under 0.5 Hz DUV modulation at temperatures from 25 to 200 °C. The color map indicates the test temperature. (B) Mean dark current as a function of temperature. (C) Photocurrent contrast extracted from the transient response as a function of temperature.

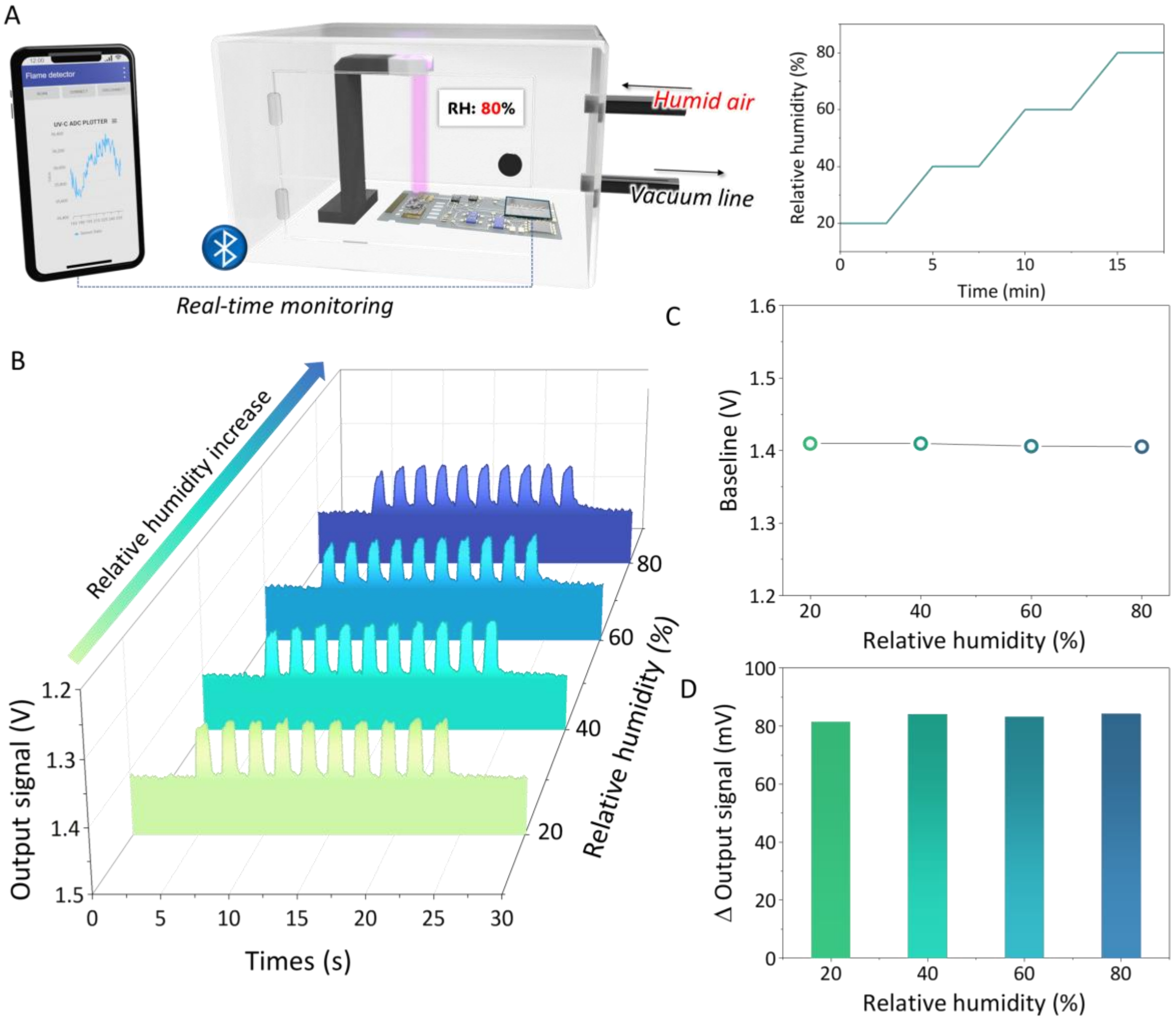

Fig. S17.

Humidity robustness evaluation of the flexible sensor-board device. (A) Schematic illustration of the humidity test setup, where the flexible sensor-board device was placed in a sealed environmental chamber and illuminated with a DUV source while the relative humidity (RH) was regulated via the gas inlet/outlet lines. (B) Transient output traces recorded under 0.5 Hz DUV modulation at different RH levels (20-80%). (C) Baseline output level in the DUV-Off state as a function of RH. (D) Output contrast (Δsignal) extracted from the transient response as a function of RH.

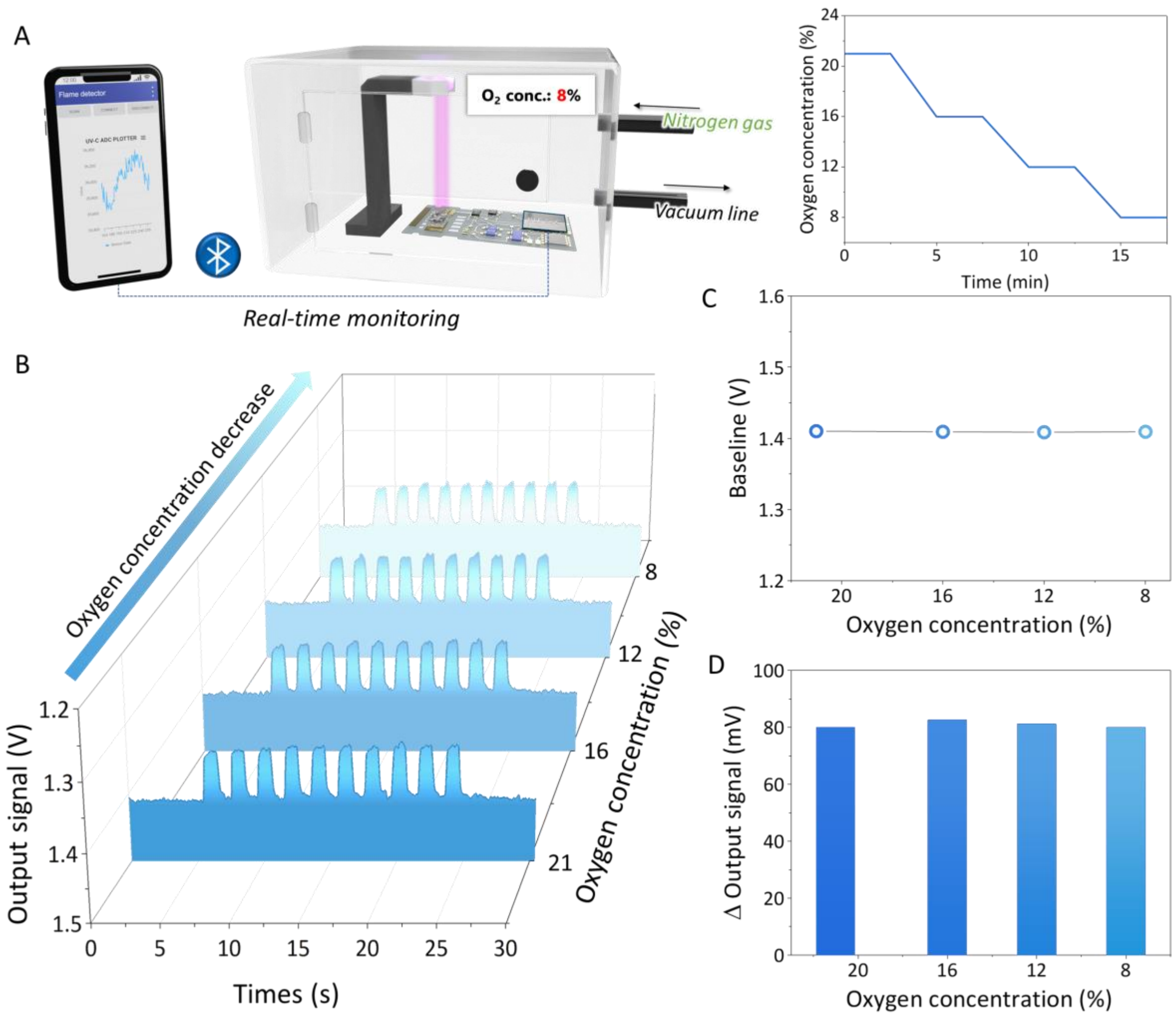

Fig. S18.

Oxygen-concentration durability evaluation of the flexible sensor-board device. (A) Schematic illustration of the oxygen-concentration test setup, where the flexible sensor-board device was placed in a controlled-atmosphere chamber and illuminated with a DUV source while the oxygen concentration was adjusted by nitrogen flow and monitored in real time. (B) Transient output traces recorded under 0.5 Hz DUV modulation at different oxygen concentrations (21 - 8%). (C) Baseline output as a function of oxygen concentration. (D) Output contrast (Δsignal) extracted from the transient response as a function of oxygen concentration.

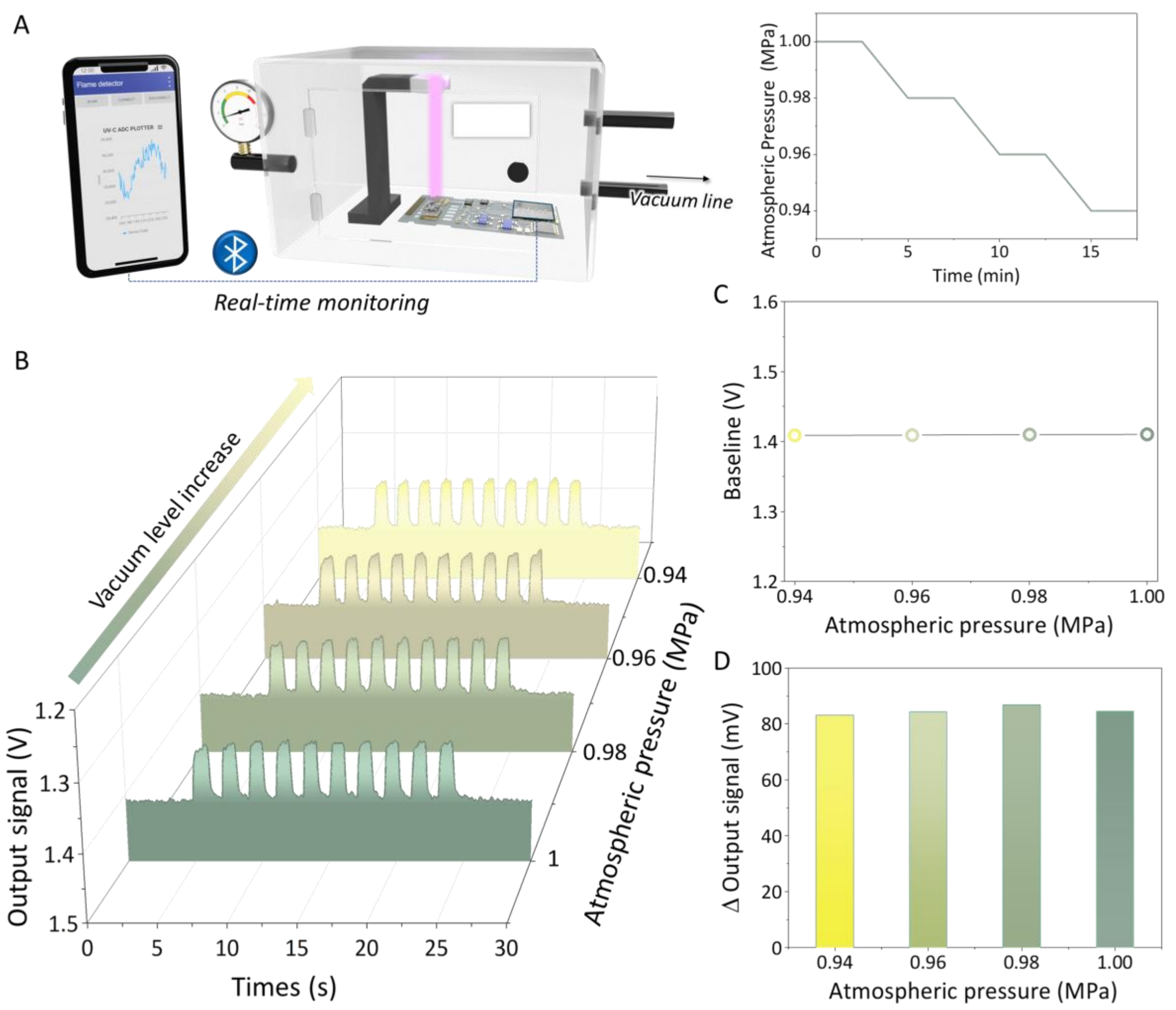

Fig. S19.

Pressure durability evaluation of the flexible sensor-board device under reduced atmospheric pressure. (A) Schematic illustration of the pressure durability test setup, where the flexible sensor-board device was placed in a chamber connected to a vacuum line and illuminated with a DUV source while the chamber pressure was monitored in real time. (B) Transient output traces recorded under 0.5 Hz DUV modulation at different atmospheric pressures (1.00 - 0.94 MPa). (C) Baseline output level in the DUV-Off state as a function of atmospheric pressure. (D) Output contrast (Δsignal) extracted from the transient response as a function of atmospheric pressure.

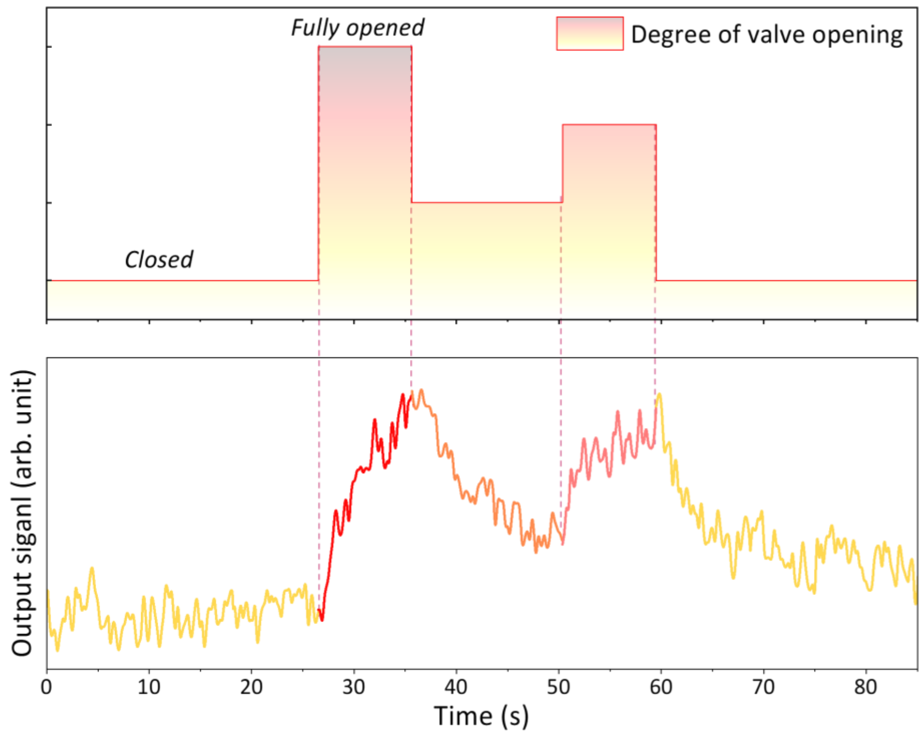

Fig. S20.

Real-time flame sensing behavior under dynamic intensity modulation. Time-resolved output signals of the circuit-integrated flexible ZTO DUV photodetector.

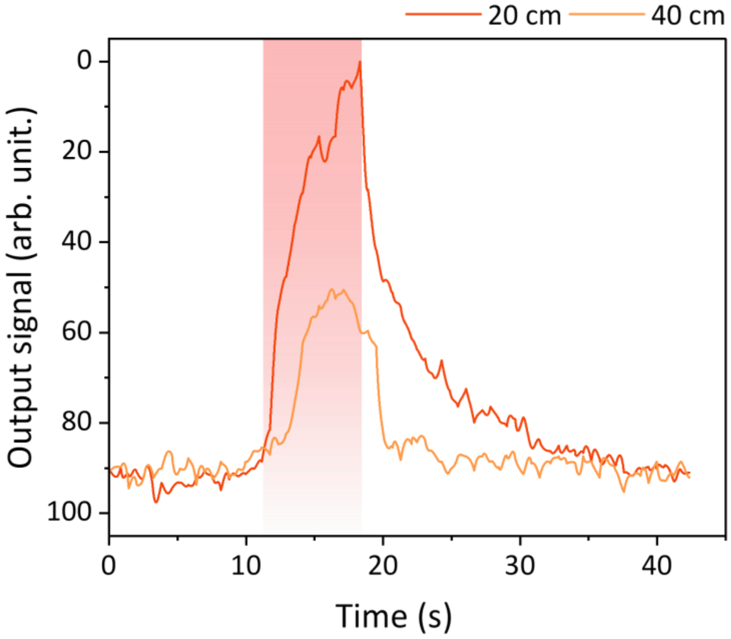

Fig. S21.

Distance-dependent flame sensing responses. Transient photoresponses of the circuit-integrated ZTO DUV photodetector as a function of flame-to-sensor distance.

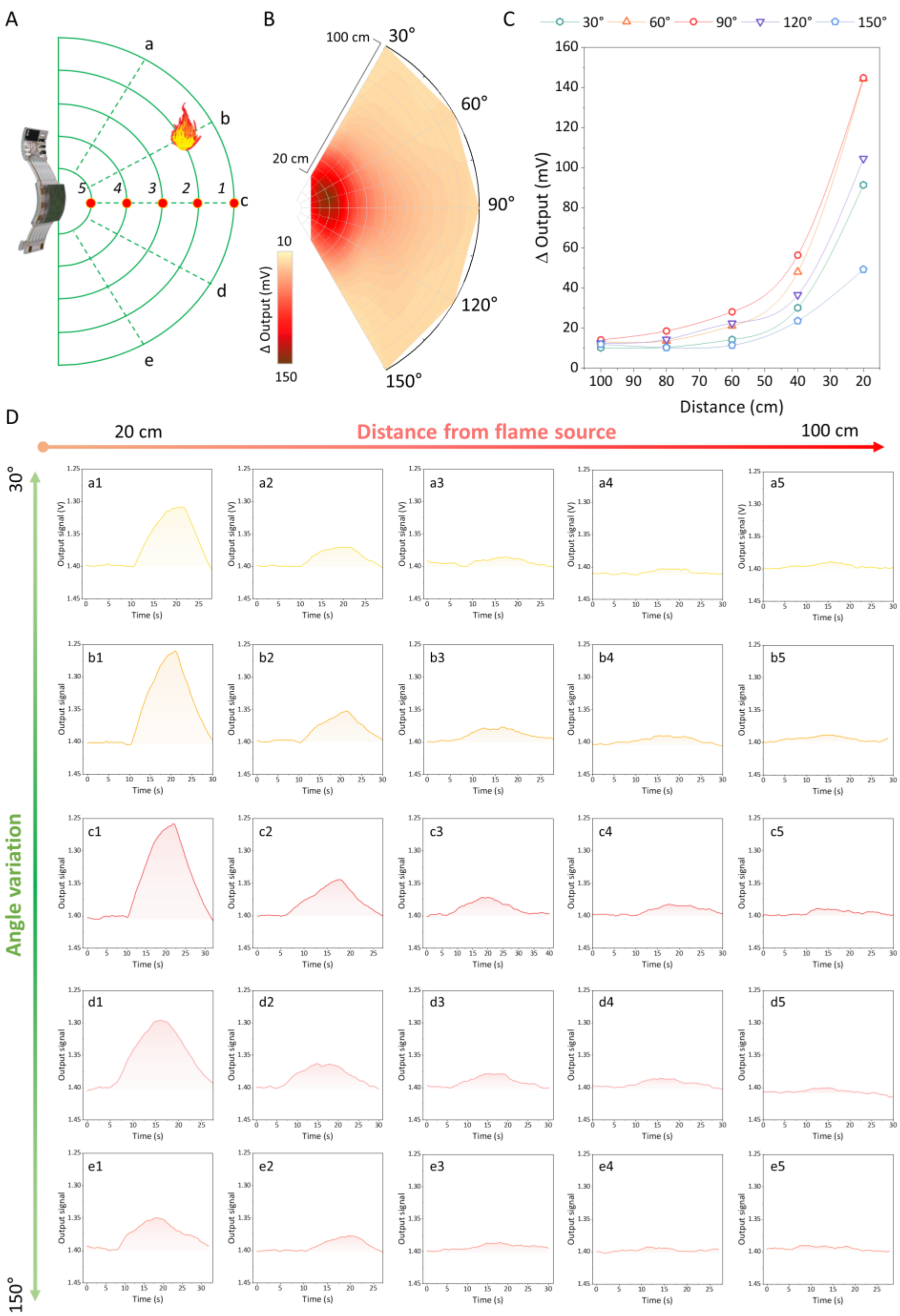

Fig. S22.

Distance- and angle-dependent flame-sensing response of the proposed device. (A) Schematic illustration of the measurement geometry used to evaluate flame detection as a function of sensor-flame distance and viewing angle. (B) Polar response map showing the relative output level as a function of angle with respect to the flame source. (C) Output intensity trend as a function of distance from the flame source for multiple angular conditions (symbols correspond to different angles). (D) Representative transient output traces measured at different distances (left to right) and viewing angles (top to bottom), illustrating the distance- and angle-dependent response characteristics.

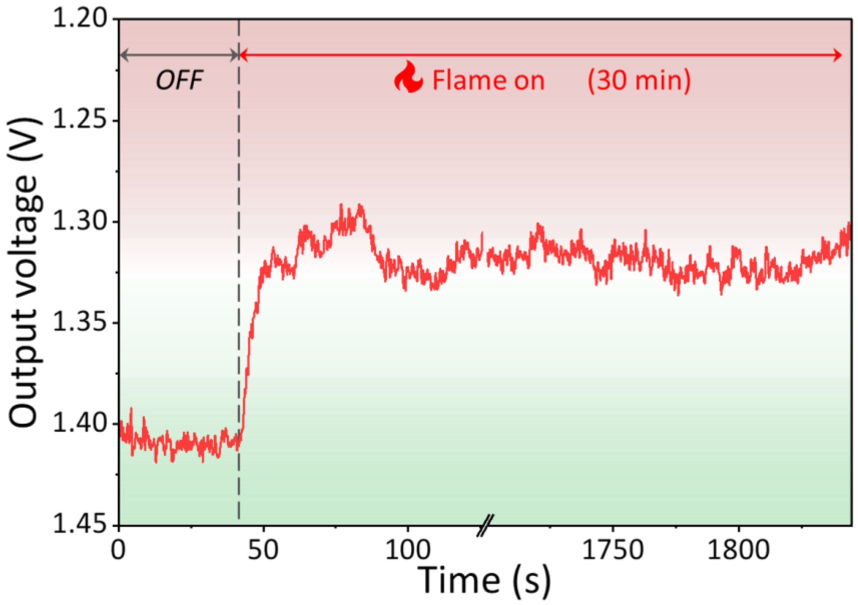

Fig. S23.

Long-duration response under a gas-stove flame source. Real-time output trace measured at a fixed flame to sensor distance of 40 cm for a gas-stove flame over 30 min.

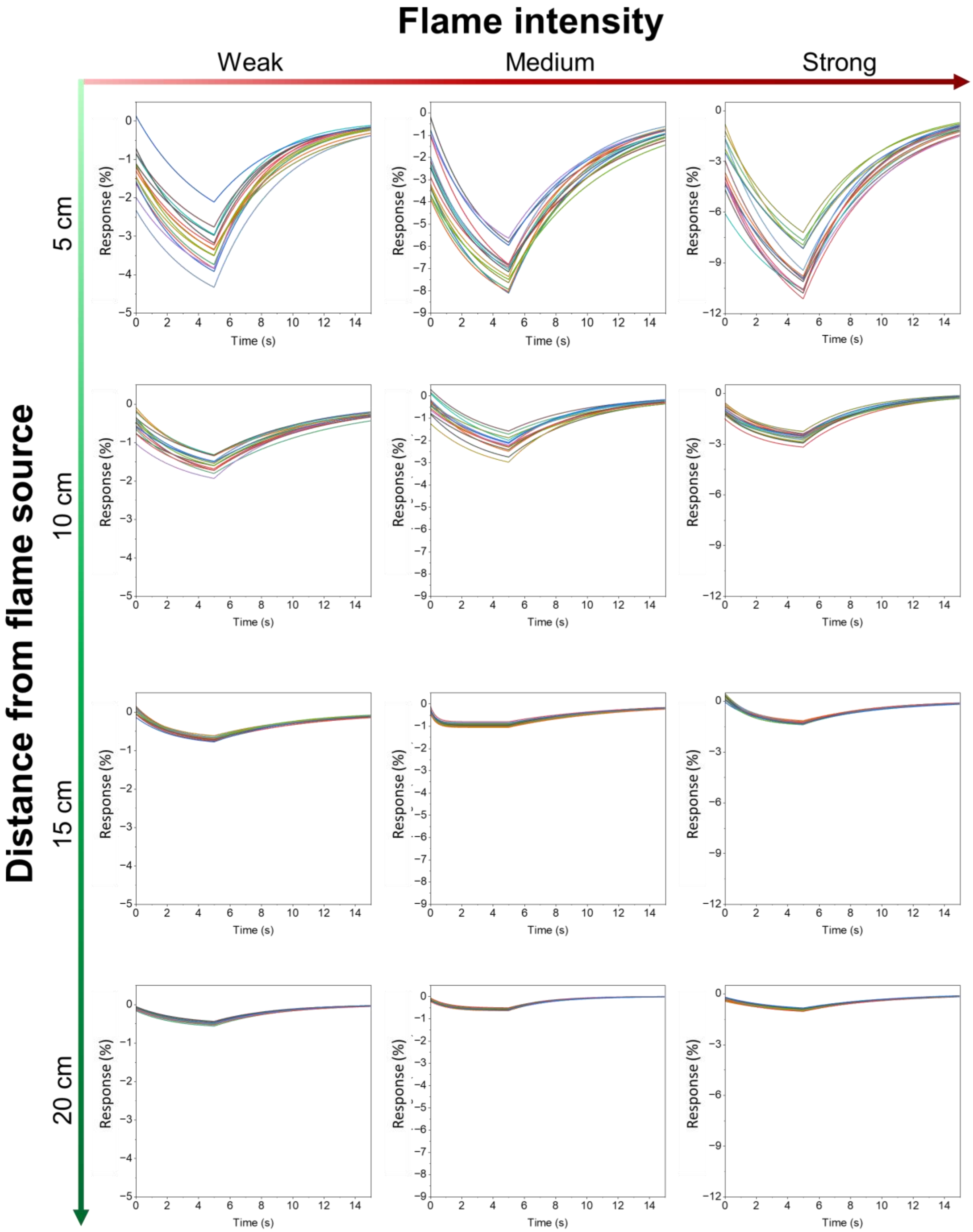

Fig. S24.

Fitted transient responses under torch flame conditions. Fitted transient response profiles of the proposed sensor system.

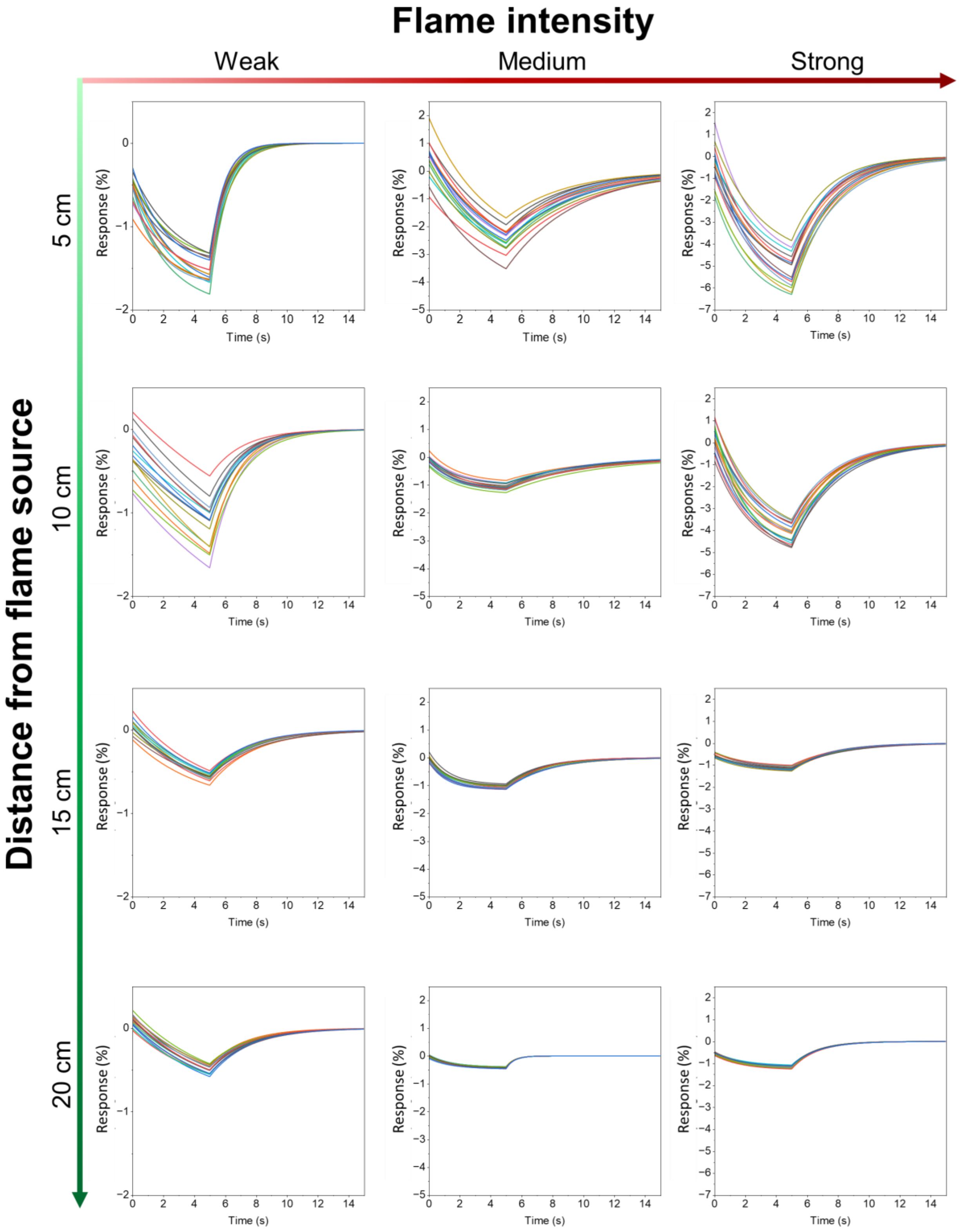

Fig. S25.

Fitted transient responses under solid-fuel flame conditions. Fitted transient response profiles of the proposed sensor system.

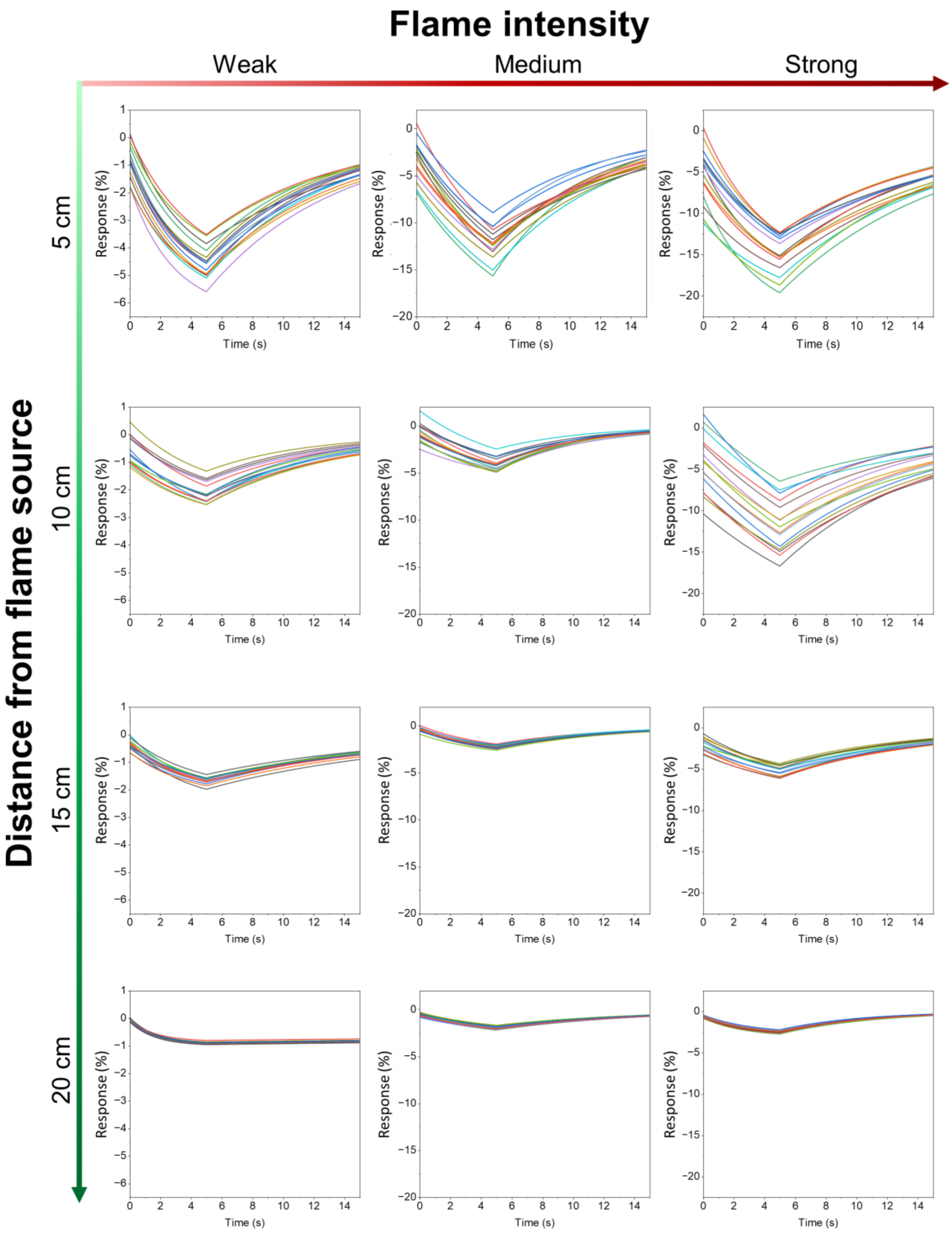

Fig. S26.

Fitted transient responses under gas stove flame conditions. Fitted transient response profiles of the proposed sensor system.

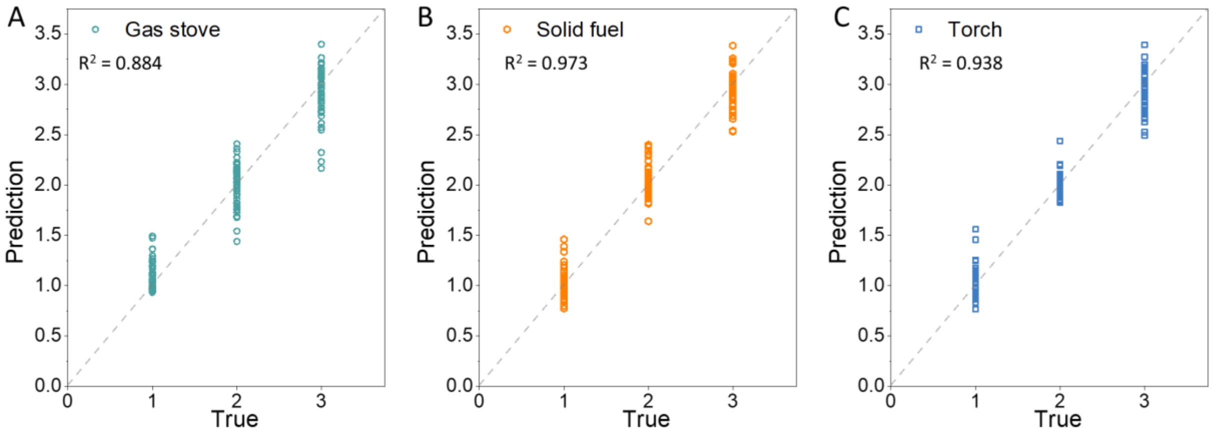

Fig. S27.

MLP-based flame intensity regression results for the training dataset. Regression outputs for (A) gas stove, (B) solid-fuel, and (C) torch flame conditions.

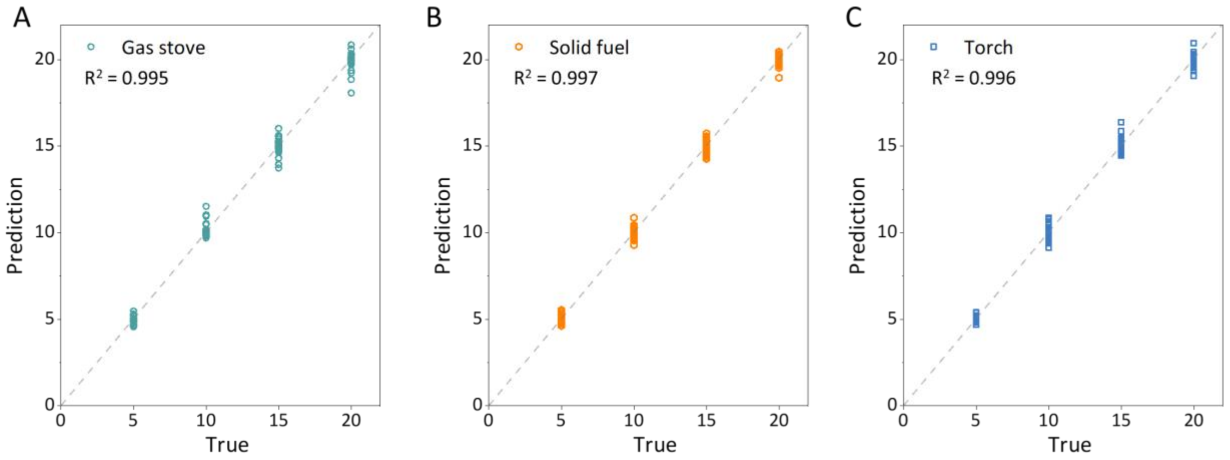

Fig. S28.

MLP-based flame distance regression results for the training dataset. Regression outputs for (A) gas stove, (B) solid-fuel, and (C) torch flame conditions.

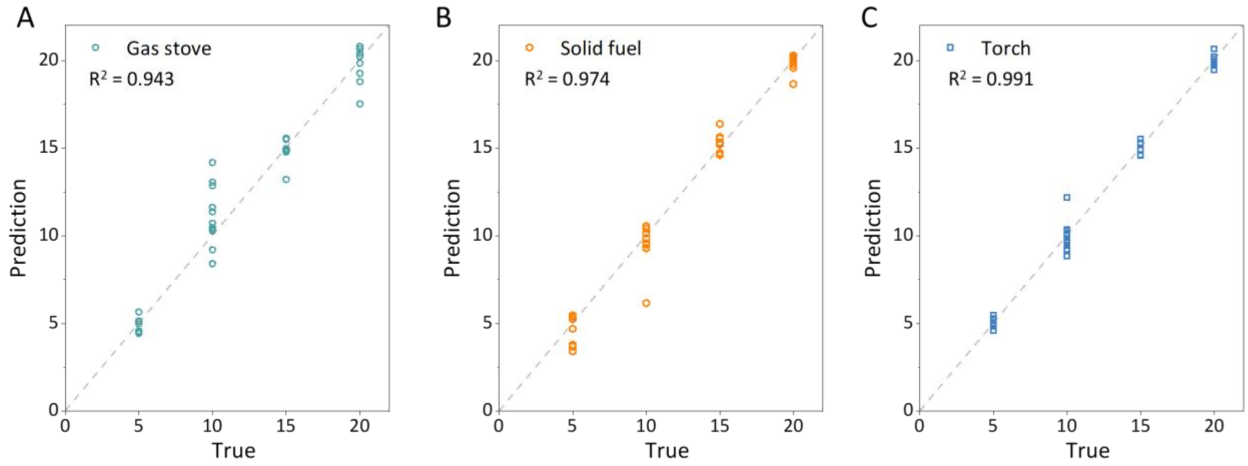

Fig. S29.

MLP-based flame distance regression results for the test dataset. Regression outputs for (A) gas stove, (B) solid-fuel, and (C) torch flame conditions.

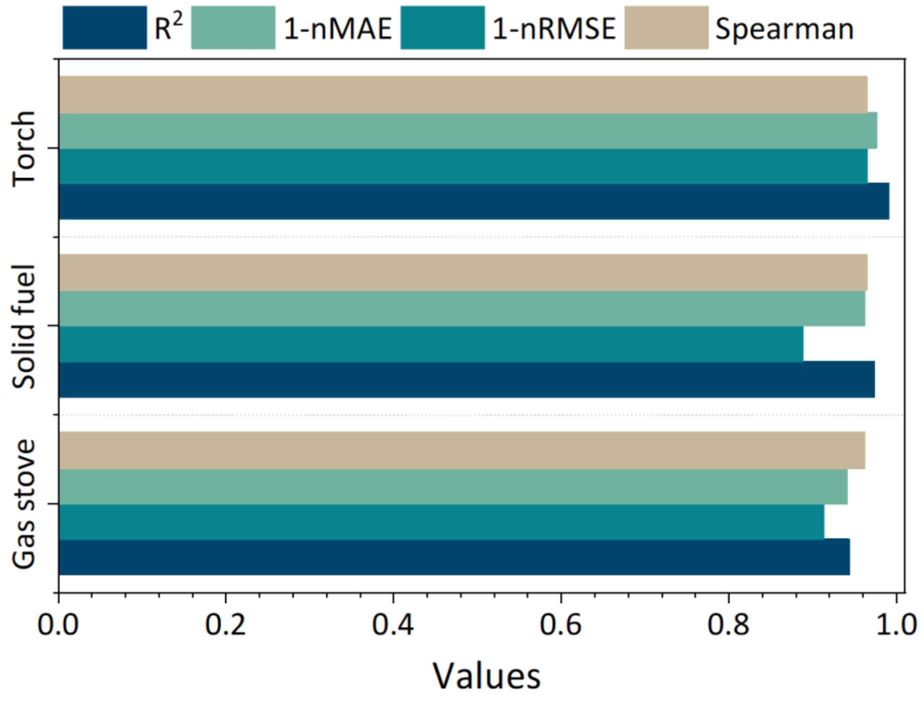

Fig. S30.

Performance metrics of flame intensity regression. Comparison of regression accuracy for gas stove, solid-fuel, and torch flame conditions.

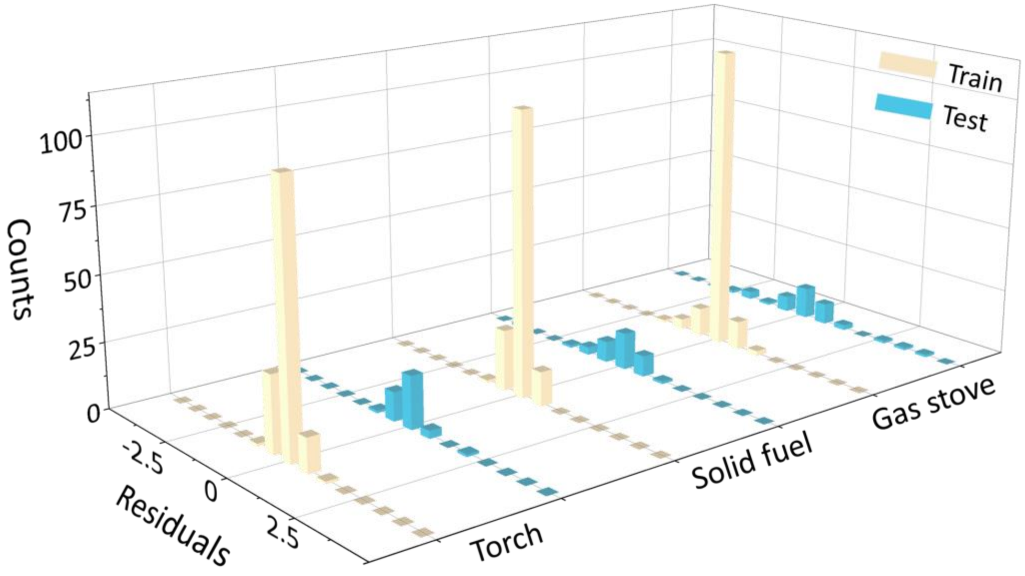

Fig. S31.

Residual distribution of flame-to-sensor distance prediction. Histogram of residuals defined as the difference between predicted and true distances.

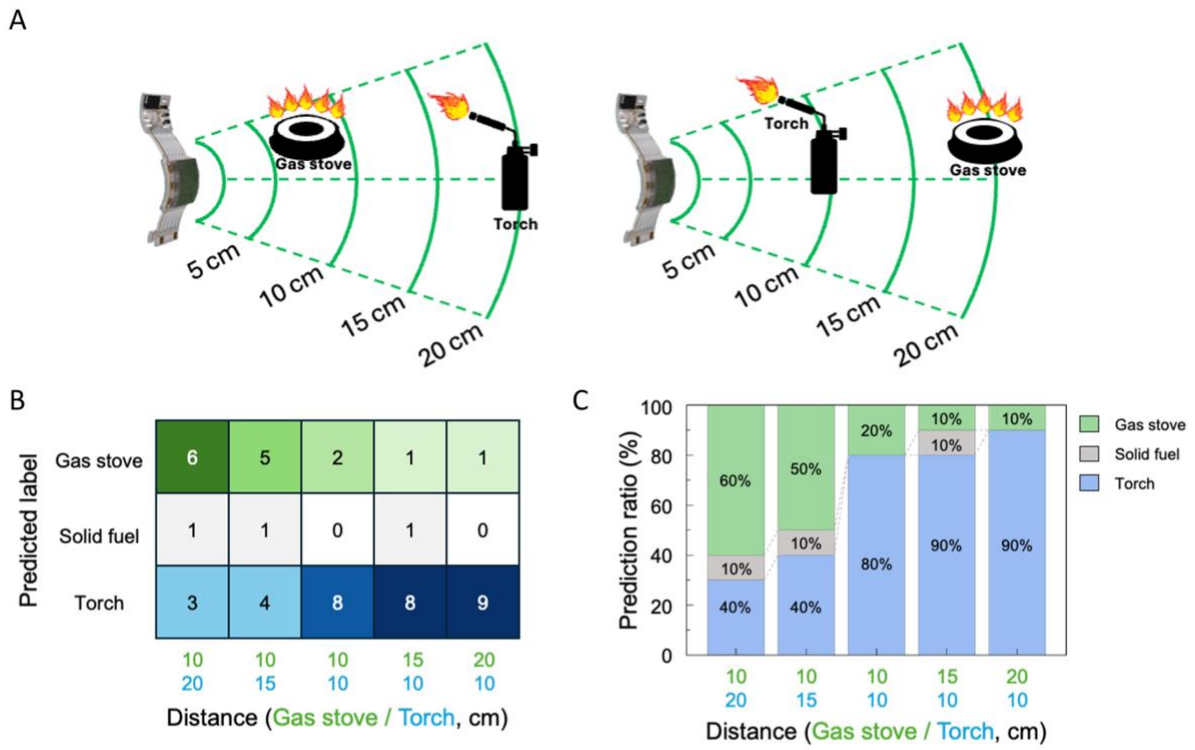

Fig. S32.

Classification outcomes under overlapping flame-source conditions using the trained MLP model. (A) Schematic illustration of the experimental environment. (B) Prediction counts (10 trials per condition) for mixed signals measured with two simultaneously active flame sources (gas stove and torch) under five overlap conditions labeled as (Gas stove / Torch) = (20/10), (15/10), (10/10), (10/15), and (10/20). (C) The corresponding prediction ratios (%) for each overlap condition, summarized as stacked bars (gas stove/solid fuel/torch).

Movie. S1.

Photoresponse under white LED and DUV pulses. Transient photoresponse under co-illumination of the white and DUV LEDs, representing DUV selectivity of the proposed device.

Movie. S2.

Real-time flame detection. Transient response of the proposed device under dynamic flame fluctuation.

REFERENCES

1. J. T. Abatzoglou, C. A. Kolden, A. C. Cullen, M. Sadegh, E. L. Williams, M. Turco, M. W. Jones, Climate change has increased the odds of extreme regional forest fire years globally. *Nat. Commun.* **16**, 6390 (2025).

2. C. X. Cunningham, G. J. Williamson, D. M. Bowman, Increasing frequency and intensity of the most extreme wildfires on Earth. *Nat. Ecol. Evol.* **8**, 1420–1425 (2024).

3. D. Drysdale, *An Introduction to Fire Dynamics* (John Wiley & Sons, 2011).

4. J. Gong, L. Yang, A review on flaming ignition of solid combustibles: Pyrolysis kinetics, experimental methods and modelling. *Fire Technol.* **60**, 893–990 (2024).

5. X. Li, A. Vázquez-López, J. Sánchez del Río Sáez, D.-Y. Wang, Recent advances on early-stage fire-warning systems: Mechanism, performance, and perspective. *Nano-Micro Lett.* **14**, 197 (2022).

6. B. Aldughayfiq, I. Alrashdi, M. A. Tawfeek, L. Jamel, W. M. Shaban, F. M. Talaat, Real-time forest fire detection, monitoring, and mitigation to safeguard humanity using YOLO and federated learning. *Int. J. Distrib. Sens. Netw.* **2025**, 4707734 (2025).

7. U. Dampage, L. Bandaranayake, R. Wanasinghe, K. Kottahachchi, B. Jayasanka, Forest fire detection system using wireless sensor networks and machine learning. *Sci. Rep.* **12**, 46 (2022).

8. G. Kuznetsov, A. Zhdanova, R. Volkov, P. Strizhak, Optimizing firefighting agent consumption and fire suppression time in buildings by forming a fire feedback loop. *Process Saf. Environ. Prot.* **165**, 754–775 (2022).

9. S. Xiao, S. Wang, L. Ge, H. Weng, X. Fang, Z. Peng, W. Zeng, Hybrid feature fusion-based high-sensitivity fire detection and early warning for intelligent building systems. *Sensors* **23**, 859 (2023).

10. J. Fonollosa, A. Solórzano, S. Marco, Chemical sensor systems and associated algorithms for fire detection: A review. *Sensors* **18**, 553 (2018).

11. E. G. Bakhoum, High-sensitivity miniature smoke detector. *IEEE Sensors J.* **12**, 3031–3035 (2012).

12. B. Liu, D. Alvarez-Ossa, N. P. Kherani, S. Zukotynski, K. P. Chen, Gamma-free smoke and particle detector using tritiated foils. *IEEE Sensors J.* **7**, 917–918 (2007).

13. D. Kozeki, "Smoldering fire detection by image processing," in *12th International Conference on Automatic Fire Detection* (National Institute of Standards and Technology, 2001), pp. 71–78.

14. N. Ya'acob, M. S. M. Najib, N. Tajudin, A. L. Yusof, M. Kassim, Image processing based forest fire detection using infrared camera. *J. Phys.: Conf. Ser.* **1768**, 012014 (2021).

15. F. Khan, Z. Xu, J. Sun, F. M. Khan, A. Ahmed, Y. Zhao, Recent advances in sensors for fire detection. *Sensors* **22**, 3310 (2022).

16. L. Deng, S. Wu, S. Zou, Q. Liu, Large-space fire detection technology: A review of conventional detector limitations and image-based target detection techniques. *Fire* **8**, 358 (2025).

17. S. Lee, T. Park, J. Hur, H. Yoo, Calcium titanate orthorhombic perovskite-nickel oxide solar-blind UVC photodetectors with unprecedented long-term stability exceeding 500 days and their applications to real-time flame detection. *ACS Photonics* **9**, 4005–4016 (2022).

18. Z. Yu, X. Qu, Y. Wan, Q. Jiang, Y. Qin, J. Xu, J. Liu, H. He, Robust and ultra-sensitive self-powered fire warning sensor based on polyimide thermoelectric fibers for temperature sensing and intelligent fire safety monitoring. *Chem. Eng. J.* **496**, 154033 (2024).

19. A. Mohapatra, T. Trinh, Early wildfire detection technologies in practice—A review. *Sustainability* **14**, 12270 (2022).

20. Y. Kong, H. Jin, G. Zhang, B. Yuan, Integration of dual fire alarm self-powered system: Leveraging intelligent wearable flame-retardant hydrophobic cotton fabric. *Chem. Eng. J.* **496**, 154158 (2024).

21. T. Park, D. H. Lee, J. Hur, H. Yoo, Unleashing the power of quantum dots: Emerging applications from deep-ultraviolet photodetectors for brighter futures. *Adv. Opt. Mater.* **12**, 2302466 (2024).

22. Z. Li, T. Yan, X. Fang, Low-dimensional wide-bandgap semiconductors for UV photodetectors. *Nat. Rev. Mater.* **8**, 587–603 (2023).

23. Y. Wang, Y. Han, Y. Wang, Y. Song, S. Fu, C. Gao, B. Li, A. Shen, Y. Liu, A graphene-enhanced PEDOT: PSS/β-Ga_2O_3 microwire organic–inorganic hybrid heterojunction self-driven photodetector with high light responsivity. *J. Mater. Chem. C* **13**, 10.1039/D5TC02594J (2025).

24. Y. Chen, Y. Li, Y. Yang, X. Yang, C. Shan, G. Shen, High-performance flexible deep ultraviolet photodetector based on crossed microwires heterojunction for real-time target trajectory tracking system. *Nano Energy* **138**, 110833 (2025).

25. X. Li, F. Xu, X. Wang, J. Luo, K. Ding, L. Ye, H. Li, Y. Xiong, P. Yu, C. Kong, Solar-blind deep-ultraviolet photoconductive detector based on amorphous Ga_2O_3 thin films for corona discharge detection. *Phys. Status Solidi RRL* **18**, 2200512 (2024).

26. X. Zhang, X. Liu, B. Sun, H. Ye, C. He, L. Kong, T. Shi, G. Liao, Z. Liu, Broadening the spectral response of perovskite photodetector to the solar-blind ultraviolet region through phosphor encapsulation. *ACS Appl. Mater. Interfaces* **13**, 44509–44519 (2021).

27. C. Zhou, J. Wang, L. Shu, J. Hu, Z. Xi, S. Li, W. Tang, ε-Ga_2O_3 solar-blind photodetector: Pyroelectric effect and flame sensing application. *Vacuum* **234**, 114060 (2025).

28. Z. Xi, Z. Liu, L. Yang, K. Tang, L. Li, G. Shen, M. Zhang, S. Li, Y. Guo, W. Tang, Comprehensive study on ultra-wide band gap La_2O_3/ε-Ga_2O_3 p–n heterojunction self-powered deep-UV photodiodes for flame sensing. *ACS Appl. Mater. Interfaces* **15**, 40744–40752 (2023).

29. Y. Wang, H. Yu, S. Li, S. Wang, C. Gao, L. Zhang, X. Cheng, Piezoelectric polarization accelerated charge separation in zinc stannate based Schottky barrier for high-efficiency hydrogen evolution. *Int. J. Hydrogen Energy* **51**, 98–110 (2024).

30. P. J. Callaghan, D. Caffrey, K. Zhussupbekov, S. Berman, A. Zhussupbekova, C. M. Smith, I. V. Shvets, Variation in the bandgap of amorphous zinc tin oxide: Investigating the thickness dependence via in situ STS. *ACS Omega* **9**, 7262–7268 (2024).

31. Z.-Y. Ma, B.-S. Zhu, Y.-H. Song, Y. Xiao, Y.-C. Yin, X.-C. Ru, G.-J. Ding, M.-Y. Zhou, M. Ge, J.-M. Hao, Y.-L. Hu, H.-B. Yao, Ultrabright and efficient deep-red perovskite light-emitting diodes enabled by ternary zinc stannate nanocrystal electron transport layer. *Adv. Opt. Mater.* **13**, 2500282 (2025).

32. Y. Jeon, J. Seo, H. Yoo, Air-stable ambipolar charge transport behaviors of organic-inorganic hybrid bilayer and application to Au nanoparticle-based floating gate memory. *J. Alloys Compd.* **938**, 168687 (2023).

33. J. Zheng, Y. Luo, X. Wen, Q. Zhang, Y. Song, J. Zhou, N. Jiang, L. Liu, F. Huang, Z. Xie, Induced crystallization of sol–gel-derived zinc oxide for efficient non-fullerene polymer solar cells. *J. Mater. Chem. A* **9**, 9616–9623 (2021).

34. S. Bandyopadhyay, G. Paul, R. Roy, S. Sen, S. Sen, Study of structural and electrical properties of grain-boundary modified ZnO films prepared by sol–gel technique. *Mater. Chem. Phys.* **74**, 83–91 (2002).

35. A. Singh, S. K. Yadav, A. Verma, S. Sikarwar, B. C. Yadav, Hydrothermally synthesized $ZnSnO_3$ nanoflakes based low-cost sensing device for high performance CO_2 monitoring. *ECS Adv.* **2**, 016501 (2023).

36. P. Pattanasattayavong, V. Promarak, T. D. Anthopoulos, Electronic properties of copper (I) thiocyanate (CuSCN). *Adv. Electron. Mater.* **3**, 1600378 (2017).

37. J. Bilbao, A. de Migue, Erythemal solar irradiance, UVER, and UV index from ground-based data in central Spain. *Appl. Sci.* **10**, 6589 (2020).

38. J. Herman, B. Biegel, L. Huang, Inactivation times from 290 to 315 nm UVB in sunlight for SARS coronaviruses CoV and CoV-2 using OMI satellite data for the sunlit Earth. *Air Qual. Atmos. Health* **14**, 217–233 (2021).

39. Y. Yang, X. Liu, Y. Wu, Review of solar spectral irradiance modelling at ground level: Current methods and machine learning opportunities. *Energy*, **344**, 139967 (2026).

40. N. Gasparini, F. V. A. Camargo, S. Frühwald, T. Nagahara, A. Classen, S. Roland, A. Wadsworth, V. G. Gregoriou, C. L. Chochos, D. Neher, M. Salvador, D. Baran, I. M. Culloch, A. Görling, L. Lüer, G. Cerullo, C. J. Brabec, Adjusting the energy of interfacial states in organic photovoltaics for maximum efficiency. *Nat. Commun.* **12**, 1772 (2021).

41. W. Kim, H. Kim, T. J. Yoo, J. Y. Lee, J. Y. Jo, B. H. Lee, A. A. Sasikala, G. Y. Jung, Y. Pak, Perovskite multifunctional logic gates via bipolar photoresponse of single photodetector. *Nat. Commun.* **13**, 720 (2022).

42. Q. Cai, H. You, H. Guo, J. Wang, B. Liu, Z. Xie, D. Chen, H. Lu, Y. Zheng, R. Zhang, Progress on AlGaN-based solar-blind ultraviolet photodetectors and focal plane arrays. *Light Sci. Appl.* **10**, 94 (2021).

43. X. Geng, Y. Liu, X. Zou, E. M. Johansson, J. Sa, Transient energy-resolved photoluminescence study of excitons and free carriers on $FAPbBr_3$ and $FAPbBr_3/SnO_2$ interfaces. *J. Phys. Chem. C* **127**, 3085–3092 (2023).

44. J. I. Khan, Y. Yang, J. R. Palmer, S. B. Tyndall, S. Chaudhuri, C. Liu, L. Grater, J. D. North, B. Chen, R. M. Young, Evaluation of interfacial photophysical processes by time-resolved optical spectroscopy in perovskite solar cells. *Matter* **7**, 2536–2550 (2024).

45. T. Park, J. Hur, Self-powered low-cost UVC sensor based on organic-inorganic heterojunction for partial discharge detection. *Small* **17**, e2100695 (2021).

46. J. Wen, Y. Zhao, P. Wu, Y. Liu, X. Zheng, R. Lin, S. Wan, K. Li, H. Luo, Y. Tian, Heterojunction formed via 3D-to-2D perovskite conversion for photostable wide-bandgap perovskite solar cells. *Nat. Commun.* **14**, 7118 (2023).

47. L. Shen, P. Song, K. Jiang, L. Zheng, J. Qiu, F. Li, Y. Huang, J. Yang, C. Tian, A. K.-Y. Jen, Ultrathin polymer membrane for improved hole extraction and ion blocking in perovskite solar cells. *Nat. Commun.* **15**, 10908 (2024).

48. W. Chai, L. Li, W. Zhu, D. Chen, L. Zhou, H. Xi, J. Zhang, C. Zhang, Y. Hao, Graded heterojunction improves wide-bandgap perovskite for highly efficient 4-terminal perovskite/silicon tandem solar cells. *Research* **6**, 0196 (2023).

49. T. M. H. Nguyen, C. W. Bark, Self-powered UVC photodetector based on europium metal-organic framework for facile monitoring invisible fire. *ACS Appl. Mater. Interfaces* **14**, 45573–45581 (2022).

50. C. Avila-Avendano, M. I. Pintor-Monroy, A. Ortiz-Conde, J. A. Caraveo-Frescas, M. A. Quevedo-Lopez, Deep UV sensors enabling solar-blind flame detectors for large-area applications. *IEEE Sensors J.* **21**, 14815–14821 (2021).

51. Y. Liu, L. X. Pang, J. Liang, M. K. Cheng, J. J. Liang, J. S. Chen, Y. H. Lai, I. K. Sou, A compact solid-state UV flame sensing system based on wide-gap II–VI thin film materials. *IEEE Trans Ind Electron* **65**, 2737–2744 (2018).

52. T. Oshima, T. Okuno, N. Arai, N. Suzuki, H. Hino, S. Fujita, Flame detection by a β-Ga_2O_3-based sensor. *Jpn. J. Appl. Phys.* **48**, 011605 (2009).

53. E. V. Gorokhov, A. N. Magunov, V. S. Feshchenko, A. A. Altukhov, Solar-blind UV flame detector based on natural diamond. *Instrum. Exp. Tech.* **51**, 280–283 (2008).

54. A. Hirano, C. Pernot, M. Iwaya, T. Detchprohm, H. Amano, I. Akasaki, Demonstration of flame detection in room light background by solar-blind AlGaN PIN photodiode. *Phys. Status Solidi* **188**, 293–296 (2001).
